# International exchange on the clinical practice and research of rheumatic skin diseases: A report of the 5th International Conference of Cutaneous Lupus Erythematosus

**DOI:** 10.1111/1346-8138.17153

**Published:** 2024-03-07

**Authors:** Minoru Hasegawa, François Chasset, Benjamin F. Chong, David F. Fiorentino, Manabu Fujimoto, Filippa Nyberg, Shinichi Sato, Joerg Wenzel, Victoria P. Werth

**Affiliations:** ^1^ Department of Dermatology University of Fukui Fukui Japan; ^2^ Faculté de médecine, Service de dermatologie et allergologie Hôpital Tenon, Sorbonne Université Paris France; ^3^ Department of Dermatology University of Texas Southwestern Medical Center Dallas Texas USA; ^4^ Department of Dermatology Stanford University School of Medicine Stanford California USA; ^5^ Department of Dermatology Osaka University Graduate School of Medicine Osaka Japan; ^6^ Department of Pathology and Department of Dermatology Karolinska University Hospital Stockholm Sweden; ^7^ Department of Dermatology, Graduate School of Medicine The University of Tokyo Tokyo Japan; ^8^ Department of Dermatology and Allergy University Hospital Bonn Bonn Germany; ^9^ Corporal Michael J. Crescenz Veterans' Administration Medical Center Philadelphia Pennsylvania USA; ^10^ Department of Dermatology, Perelman School of Medicine University of Pennsylvania Philadelphia Pennsylvania USA

## Abstract

The 5th International Conference of Cutaneous Lupus Erythematosus was held in Tokyo, Japan on May 9 and 10, 2023. The latest topics on the pathogenesis, diagnosis, assessment, and treatment of cutaneous lupus erythematosus, dermatomyositis, and scleroderma (systemic sclerosis, morphea) were presented by experts in each field and new developments discussed. In these rheumatic skin diseases, many clinical trials of novel therapies targeting cytokines, signaling molecules, plasmacytoid dendritic cells, B cells, and other molecules are currently underway, and standardization of outcome assessment was discussed. In addition, the selection of the therapeutic agents available for the diversity of each case is becoming more important, together with the ongoing pathophysiological analysis of the diseases. The achievements of this conference will further promote the development of clinical practice and research in rheumatic skin diseases through international exchange among researchers. We hope that by reporting a summary of the conference in this manuscript, we can share its contents with readers.

The 5th International Conference of Cutaneous Lupus Erythematosus (ICCLE) was held on May 9 (Tuesday) and 10 (Wednesday), 2023, at the Ito International Research Center, University of Tokyo, Japan. This conference has been held in conjunction with International Investigative Dermatology (now International Societies for Investigative Dermatology), which has been held every 5 years since 2004 as a research meeting for cutaneous lupus erythematosus (CLE). The first ICCLE was hosted by Dr. Annegret Kuhn in Dusseldorf, Germany,^1^ and the second meeting was held by Dr. Fukumi Furukawa in Kyoto, Japan.^2^ Both the third meeting (Edinburgh, Scotland)^3^ and the fourth meeting (Orlando, USA)^4^ were organized by Dr. Victoria Werth. From the third conference, not only CLE but also dermatomyositis (DM) was included in the scope. During this period, the international collaboration has contributed greatly to the development of clinical and/or basic research on CLE and DM, including pathogenesis, epidemiology, genetics, diagnosis, assessment, and treatment.^5^, ^6^

This 5th meeting was organized just before the International Society for Investigative Dermatology 2023 (president, Dr. Kenji Kabashima), held May 10–14 at the Keio Plaza Hotel in Tokyo, Japan. Of special note, a new session on scleroderma, consisting of systemic sclerosis (SSc) and morphea, was added to the program. The international program committee members were Drs. Minoru Hasegawa (host and chair, Japan), Francois Chasset (France), Benjamin Chong (USA), David Fiorentino (USA), Manabu Fujimoto (host, Japan), Filippa Nyberg (Sweden), Shinichi Sato (host, Japan), Joerg Wenzel (Germany), and Victoria Werth (USA). An overview of the 2‐day program follows. Abstracts of lectures and presentations are provided as Data S1.

## 1 DAY 1 (MAY 9, TUESDAY)

### 1.1 Opening

Following the opening remarks by Dr. Minoru Hasegawa, Dr. Victoria Werth introduced the history of ICCLE, its past activities, and its future plans.

### 1.2 Pathogenesis and clinical insights of scleroderma

Dr. Heidi Jacobe (USA): Dr. Jacobe reviewed a large number of morphea cases, ranging from mild disease to severe disease with extracutaneous lesions, and found that activity usually resolves within a year with standard treatment, but some cases relapse, and the course and outcome of symptoms are related to disease type and depth of lesions.

Dr. Takemichi Fukasawa (Japan): Dr. Fukasawa reported that B cells that respond to topoisomerase I with high affinity produce cytokines such as IL‐6 and IL‐23, and induce the differentiation of T cells into Th17 cells, which are involved in fibrosis in a mouse model of SSc and patients with SSc.

Dr. Ayumi Yoshizaki (Japan): Dr. Yoshizaki et al. have established a method for comprehensive detection of autoantibodies in serum using proprietary technology of transcriptome‐wide comprehensive autoantibody assay. The assay is already available on a commercial basis with high sensitivity and specificity for SSc and DM under the name “A‐cube”.

### 1.3 Basic and clinical studies of rheumatic diseases (selected from open submissions)

Dr. Ai Kuzumi (Japan): Long‐term efficacy of rituximab in patients with systemic sclerosis: follow‐up of the DESIRES trial with a focus on immunoglobulin levels. Dr. Kuzumi noted that long‐term rituximab treatment for SSc has been shown to be beneficial for skin sclerosis and ILD of SSc, and that serum immunoglobulin levels may be useful in assessing and predicting efficacy.

Dr. Megan Zhao (USA): Updates on the current state of management and assessment of the highlighted risk for atherosclerotic cardiovascular events in an established cohort of patients with lupus erythematosus: a multifactorial issue. Dr. Zhao assessed the management of atherosclerotic cardiovascular disease risk in a single center and indicated that hyperlipidemia may not be adequately treated.

Dr. Christopher Richardson: Lack of cutaneous B cells differentiates lupus‐like disease in MRL/LPR mice from human discoid lupus. B‐cell infiltration is prominent in human discoid lupus erythematosus (DLE). However, Dr. Richardson reported that MRL/lpr mice, a typical mouse model of SLE, is not a suitable model for DLE because B cells are rare in the skin.

Dr. Mariko Ogawa‐Momohara (USA): Multiplexed mass cytometry of cutaneous lupus erythematosus and dermatomyositis skin. Using imaging mass cytometry on CLE and DM skin samples, Dr. Ogawa‐Momohara found that the primary regulatory B cells in the skin in these diseases are plasma cells, and that CD20^+^ B cells and conventional DC (cDC) are increased in DM, whereas IL‐10‐producing plasma cells are increased in CLE. The results showed that regulatory plasma cells were increased in DM but their function may be impaired.

Dr. Victoria Werth (USA): The Total Improvement Score (TIS) in a phase 3 clinical trial for dermatomyositis (DM): Room for improvement? The Total Improvement Score (TIS), a composite score for DM, is somewhat less sensitive to skin changes, but a new, simpler composite named Dermatomyositis Outcomes for Muscle and Skin (DMOMS) Outcomes, which includes components of the TIS and skin‐specific outcomes, may be suitable for detecting treatment effects in DM clinical trials that include patients of all DM phenotypes, even with small sample sizes.

### 1.4 Pathogenesis of CLE

Dr. Joerg Wenzel (Germany): Dr. Wenzel reviewed the pathogenesis of CLE, noting that the complex interplay of interferon (IFN)‐related inflammation and acquired immunity induces CLE lesions, and that DNA/RNA damage and inadequate nuclear debris clearance are involved. He explained that there are still many unknowns to consider, including B cells with prominent infiltration in DLE.

Dr. Joerg Wenzel (Germany): Immunohistochemistry of skin lesions does not neatly stratify the pattern of predominant infiltrating cells according to CLE type, and even within the same disease type, there is diversity from case to case, suggesting treatment strategies tailored to the pathology of individual cases.

Dr. Joseph Merola (USA): A non‐invasive study using tape stripping to collect mRNA in skin lesions of CLE identified two clusters of genes, an IFN‐related gene cluster and a CLE‐related gene cluster, which correlated well with punch biopsy specimens.

### 1.5 Special lecture

Dr. Keishi Fujio (Japan): Functional genome analysis in the immune cells of SLE revealed the importance of the mitochondrial pathway in SLE. Furthermore, there are two distinct signatures in SLE, the disease‐states signature and the disease‐activity signature. Dr. Fujio suggested that the combination of such a functional genome database and a clinical cohort is important.

### 1.6 Clinical insights of CLE

Dr. Benjamin Chong (USA): Approximately 20% of patients with CLE will progress to SLE, and risk factors include ANA positivity, generalized DLE, and higher baseline number of lupus criteria.

Dr. Joseph Merola (USA): To remove barriers to clinical trial approval of CLE, Dr. Merola et al. developed the Cutaneous Lupus Activity Investigator Global Assessment (IGA) – Revised, an IGA‐based tool to measure CLE disease activity. The instrument is now being used as the primary, secondary, and exploratory endpoints in multiple phase 2/3 trials and registries.

Dr. Victoria Werth (USA): Imaging mass cytometry revealed that phosphorylation of a stimulator of interferon genes (STING) and enhanced expression of IFN‐kappa produced by cDC may be important in the pathogenesis of quinacrine‐responsive CLE. IFN‐α may be produced by keratinocytes, cDCs, macrophages rather than plasmacytoid DCs (pDCs) in CLE.

### 1.7 Treatment of CLE

Dr. François Chasset (France): Dr. Chasset discussed recent findings related to antimalarials in CLE and suggested that a cut‐off of 5 mg/kg/day of hydroxychloroquine (HCQ) is a safe range for ocular toxicity, but that this dose has been associated with increased flares in moderate to severe SLE. He stated that a different approach to the optimal dosing of HCQ is needed, such as monitoring HCQ concentrations in peripheral blood.

Dr. François Chasset (France): Dr. Chasset reported that thalidomide and lenalidomide are useful in CLE when antimalarials are ineffective.

Dr. Victoria Werth (USA): Update on CLE clinical trials. CLE targeting type I IFNs [anti‐IFNAR receptor (anifrolumab), TYK2 inhibitor (deucravacitinib)] or pDCs [(litifilimab, daxdilimab)] demonstrated good results and development is continuing in further clinical trials.

## 2 DAY 2 (MAY 10, WEDNESDAY)

### 2.1 Poster award ceremony

The following five posters received awards:

- Association between periungual changes and myositis‐specific autoantibodies in patients with idiopathic inflammatory myopathies
  Ikuko Ueda‐Hayakawa, et al. (Osaka University Graduate School of Medicine, Japan)
- Clinical features of patients with systemic sclerosis positive for anti‐SS‐A antibody: A cohort study of 156 patients
  Tomoya Watanabe, et al. (Yokohama City University, Japan)
- Factors associated with diagnostic delays in dermatomyositis
  Lindsey Wanberg, et al. (University of Minnesota Medical School, USA)
- Multiplexed mass cytometry of cutaneous lupus erythematosus and dermatomyositis skin
  Mariko Ogawa‐Momohara, et al. (Corporal Michael J. Crescenz VAMC, USA; University of Pennsylvania, USA; Nagoya University Graduate School of Medicine, Japan)
- Staphylococcus aureus skin colonization promotes SLE‐like autoimmune inflammation via neutrophil activation and the IL‐23/IL‐17 axis
  Hitoshi Terui, et al. (Tohoku University Graduate School of Medicine, Japan)

### 2.2 Pathogenesis of DM

Dr. Naoko Okiyama (Japan): Dr. Okiyama established a mouse model of DM caused by autoimmunity to transcription intermediary factor (TIF)‐1γ and melanoma differentiation‐associated gene (MDA)‐5, antigens recognized by myositis‐specific autoantibodies.

Dr. David Fiorentino (USA): TIF‐1γ, the “cancer antigen”, is often not associated with cancer emergence in DM patients in the USA. Cell division cycle and apoptosis regulator 1 (CCAR1), SP4, and other antigens confined to anti‐TIF1γ subset are associated with cancer protection.

Dr. Noriko Arase (Japan): Cellular misfolded protein/MHC class II complexes are possible autoantibody targets and Ro52/IgG/HLA‐DR complex antibodies may be involved in the pathogenesis of DM.

### 2.3 Clinical insights in dermatomyositis

Dr. Manabu Fujimoto (Japan): Dr. Fujimoto reported that autoantibodies found in DM are associated with characteristic skin manifestations and are useful in predicting the pathogenesis of DM, and that novel autoantibodies associated with DM, such as anti‐CCAR1, anti‐SP4, IgM anti‐ angiotensin converting enzyme‐2, anti‐cortactin, and anti‐Four and a half LIM domains 1 (FHL1), have been found.

Dr. Benjamin Chong (USA): In a single‐center study, DM patients with skin of color tended to have younger, more classic‐type DM, less linear extensor erythema, and more severe and chronic disease than other DM patients without skin of color.

### 2.4 Treatment of dermatomyositis

Dr. David Fiorentino (USA): Dr. Fiorentino explained that various clinical trials targeting T cells, B cells, pDC, autoantibodies, complement, JAK, type I IFN, etc. are ongoing or under investigation in DM.

Dr. Victoria Werth (USA): A phase III trial of lenabasum in DM, including amyopathic DM, failed to meet its primary endpoint. In cases with active skin disease but normal muscle strength, treatment with lenabasum resulted in significant improvement in Cutaneous Dermatomyositis Disease Area and Severity Index (CDASI) activity scores.

### 2.5 Rheum‐Derm grand rounds

Organized by Dr. Lauren Graham (USA) and Dr. Filippa Nyberg (Sweden), the following six cases were presented and actively discussed.

1. Linear cutaneous lupus erythematosus – an important differential for blaschkoid dermatoses
  Dr. Ysabel Regina Ortiz et al. (Philippines)
2. Improvement of systemic sclerosis‐associated digital ulcers after UVA1 phototherapy
  Dr. Lauren Graham et al. (USA)
3. Paraneoplastic interface dermatitis presenting as exfoliative erythroderma
  Dr. Lauren Graham et al. (USA)
4. ACLE in a young woman after dapsone treatment
  Dr. Filippa Nyberg et al. (Sweden)
5. Dermatomyositis with minimal myositis and Mi2 autoantibodies
  Dr. Filippa Nyberg et al. (Sweden)
6. Cutaneous lupus erythematosus after nivolumab treatment
  Dr. Filippa Nyberg et al. (Sweden)

### 2.6 Closing

Dr. Filippa Nyberg and Dr. Joerg Wenzel delivered the closing remarks.

### 2.7 Co‐sponsored seminars

The following representative Japanese immunologists (Dr. Sachiko Miyake and Dr. Susumu Nakae), rheumatologist (Dr. Yohei Kirino), and dermatologists (Dr. Yoshihide Asano, Dr. Masatoshi Jinnin, Dr. Takashi Matsushita, Dr. Sei‐chiro Motegi, and Dr. Rei Watanabe) gave co‐sponsored seminar lectures.

The conference was attended by over 120 participants from Japan and abroad, and 50 posters were presented. It was a valuable opportunity for international exchange about skin manifestations of rheumatic diseases. In particular, we believe that it was a stimulating experience for young Japanese dermatologists. Support was provided by the Rheumatologic Dermatology Society, the International Societies for Investigative Dermatology, the Japanese Dermatological Association, and the Japanese Society of Investigative Dermatology in organizing this conference. Finally, we would like to express our sincere gratitude to the program committee members, chairpersons, presenters, participants, co‐sponsors, convention vendors, and all those who cooperated in holding this conference, including Dr. Noritaka Oyama, secretary general. We hope that the activities of the ICCLE will lead to further research and therapeutic advances in rheumatic skin diseases.

## CONFLICT OF INTEREST STATEMENT

None declared.

## Supporting information


Data S1.
