# Supplementary material for: International exchange on the clinical practice and research of rheumatic skin diseases: A report of the 5th International Conference of Cutaneous Lupus Erythematosus
Source: J Dermatol. 2024 Mar 7;51(7):881–4. doi: 10.1111/1346-8138.17153 (PMC11483916; doi:10.1111/1346-8138.17153)
Supplement: Supplementary file 1 — Data S1. [file JDE-51--s001.pdf]

# 5th International Conference on Cutaneous Lupus Erythematosus 2023 (ICCLE 2023)

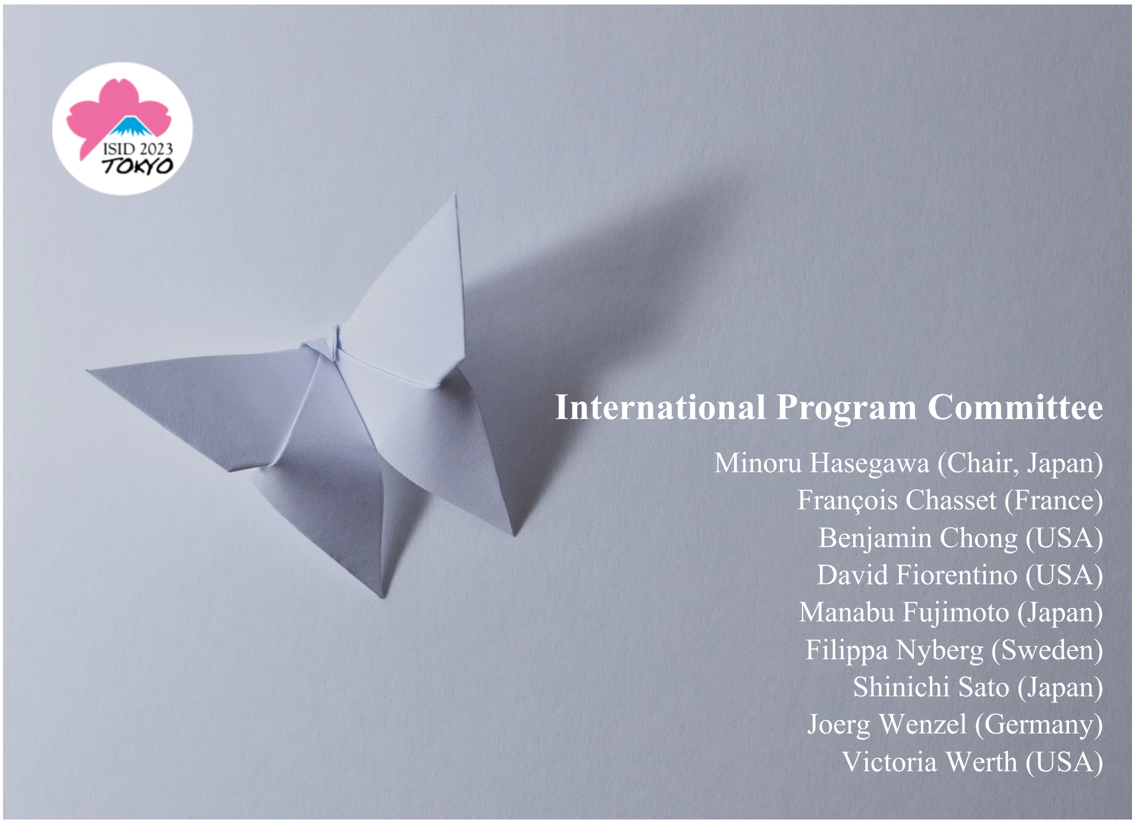


Ito International Research Center of the University of Tokyo
7-3-1 Hongo, Bunkyo-ku, Tokyo, Japan

https://www.rheumaderm-society.org/iccle-meeting/

# Timetable

## Day 1 (May 9, Tuesday)

**8:30** **Registration, putting up posters**

**9:15 - 9:20** **Opening Remarks**
Minoru Hasegawa
Department of Dermatology, University of Fukui, Japan

**9:20 - 9:25** **ICCLE: a dynamic international collaborative group to improve outcomes in autoimmune skin diseases**
Victoria Werth[1,2]
[1]Department of Dermatology, CMCVAMC, [2]Department of Dermatology, University of Pennsylvania, USA

**Session I:** **Pathogenesis and Clinical Insights of Scleroderma**

**Chair:** Heidi Jacobe and Shinichi Sato

**9:25 - 9:40** **1. Outcomes research in morphea**
Heidi Jacobe
Department of Dermatology, UT Southwestern Medical Center, USA

**9:40 - 9:55** **2. The role of B cells in systemic sclerosis**
Takemichi Fukasawa, Ayumi Yoshizaki, Asako Yoshizaki, Shinichi Sato.
Department of Dermatology, University of Tokyo Graduate School of Medicine, Japan

**9:55 - 10:05** **3. Novel comprehensive detection method for autoantibodies and its clinical application**
Ayumi Yoshizaki
Department of Dermatology, University of Tokyo Graduate School of Medicine, Japan

**10:05 - 10:15** **Discussion**

**10:15 - 10:25** **Break**

**Session II:** **Basic and Clinical Studies of Rheumatic Diseases**

**Chair:** Christopher Richardson and Yukie Yamaguchi

**10:25 - 11:15**

**1 (RO-P1)** **Long-term efficacy of rituximab in patients with systemic sclerosis: follow-up of the DESIRES trial with a focus on immunoglobulin levels**
Ai Kuzumi, Satoshi Ebata, Takemichi Fukasawa, Shinichi Sato, Ayumi Yoshizaki.
Department of Dermatology, Graduate School of Medicine, The University of Tokyo, Japan

**2 (RO-P2)** **Updates on the current state of management and assessment of the highlighted risk for atherosclerotic cardiovascular events in an established cohort of**

**patients with lupus erythematosus: a multifactorial issue**

Megan Zhao, BA[1,2], Rui Feng, PhD[3], Kevin Jon Williams, MD[4*], Victoria P. Werth, MD[1,2*]. [1]Corporal Michael J. Crescenz Veterans' Administration Medical Center, Philadelphia, PA, USA; [2]Department of Dermatology, University of Pennsylvania, Perelman School of Medicine, Philadelphia, PA; [3]Department of Biostatistics, University of Pennsylvania, Perelman School of Medicine, Philadelphia, PA; [4]Department of Cardiovascular Sciences, Department of Medicine, Lewis Katz School of Medicine at Temple University, Philadelphia, PA, USA. *Co-senior authors

3 (RO-P3) **Lack of cutaneous B cells differentiates lupus-like disease in MRL/LPR mice from human discoid lupus**

Tim Curran[1], Joshuah Yon[2], Christopher T. Richardson[1]. [1]Dermatology, University of Rochester Medical Center, Rochester, NY, United States, [2]University of Rochester, Rochester, NY, United States

4 (RO-P4) **Multiplexed mass cytometry of cutaneous lupus erythematosus and dermatomyositis skin**

Mariko Ogawa-Momohara[1,2,3] Thomas Vazquez[1,2], Meena Sharma[1,2], Joshua Dan[1,2], Grant Sprow[1,2], Victoria P. Werth[1,2]. [1]Corporal Michael J. Crescenz VAMC, Phil, PA, 19104; [2] Dermatology, University of Pennsylvania, Phil, PA, USA; [3] Dermatology, Nagoya University Graduate School of Medicine, Nagoya, Japan

5 (RO-P5) **The total improvement score (TIS) in a phase 3 clinical trial for dermatomyositis (DM): Room for improvement?**

J Dan[1,2], D Lim[1,2], R Pandya,[1,2] J Concha[1,2], J Kleitsch,[1,2] G Sprow[1,2], N Kodali[1,2], D Diaz[1,2], B White[3], VP Werth[1,2]. [1]Dermatology, PSOM, UPenn, Phil, PA, USA; [2]CMC VAMC, Phil, PA, USA; [3]Corbus Pharmaceuticals, Norwood, MA, USA

**Poster session I (Odd-numbered abstracts)**

**11:15 - 11:55**

**Sponsored Luncheon Seminar I <Janssen>**

**Chair:** Manabu Fujimoto and Victoria Werth

**12:00 - 12:30** 1. **Treatment management for digital ulcers in systemic sclerosis ~Role of bosentan in the treatment of DUs in SSc~**

Sei-ichiro Motegi
Department of Dermatology, Gunma University, Japan

**12:30 - 13:00** 2. **Diagnosis and treatment for systemic sclerosis-associated pulmonary arterial hypertension**

Takashi Matsushita
Department of Dermatology, Kanazawa University, Japan

**Session III: Pathogenesis of CLE**

**Chair:** Satoru Arai and Benjamin Chong

**13:05 - 13:20** 1. **Overview**

Joerg Wenzel
University Hospital Bonn, Germany

**13:20 - 13:35** **2. (Immuno-) histological stratification of CLE patient for targeted therapies**
Joerg Wenzel
University Hospital Bonn, Germany

**13:35 - 13:50** **3. Tape stripping: A novel, non-invasive method for identifying and measuring cutaneous lupus activity**
Joseph Merola
Harvard Medical School, Brigham and Women's Hospital, Boston, MA, USA

**13:50 - 14:00** **Discussion**

**Session IV: Special Lecture**

**Chair:** Victoria Werth

**14:00 - 14:30** **Identification of pathological pathways of SLE from the immune cell transcriptome**
Keishi Fujio
Department of Allergy and Rheumatology, Graduate School of Medicine, The University of Tokyo, Japan

**14:30 - 14:50** **Photo, Break (20 min)**

**Session V: Clinical Insights of CLE**

**Chair:** François Chasset and Hee Joo Kim

**14:50 - 15:00** **1. The relationship between systemic lupus erythematosus and cutaneous lupus erythematosus**
Benjamin F. Chong, MD, MSCS
Department of Dermatology, University of Texas Southwestern Medical Center, Dallas, TX, USA

**15:00-15:25** **2. Outcome measures in cutaneous lupus studies**
Joseph Merola
Harvard Medical School, Brigham and Women's Hospital, Boston, MA, USA

**15:25-15:40** **3. Heterogeneity of response to antimalarials and type I interferons related to dendritic cells in CLE**
Victoria P. Werth[1,2], DeAnna Diaz[1,2], Felix Chin[1,2], Meena Sharma[1,2], Thomas Vazquez[1,2], Jay Patel[1,2]
[1]Dermatology, CMCVAMC, [2]Department of Dermatology, University of Pennsylvania, USA

**15:40 - 15:50** **Discussion**

**Session VI: Treatment of CLE**

# Timetable

**Chair:** Joseph Merola and Filippa Nyberg

**15:50 - 16:05** 1. **Recent findings about antimalarial in cutaneous lupus erythematosus**
François Chasset
Sorbonne Université, Faculté de médecine, Service de dermatologie et allergologie, Hôpital Tenon, France

**16:05 - 16:20** 2. **Refractory cutaneous lupus: Use of thalidomide, lenalidomide and lenalidomide for refractory lupus skin disease**
François Chasset
Sorbonne Université, Faculté de médecine, Service de dermatologie et allergologie, Hôpital Tenon, France

**16:20 - 16:35** 3. **Update on clinical trials for cutaneous lupus erythematosus**
Victoria P. Werth[1,2]
[1]Dermatology, CMCVAMC, [2]Department of Dermatology, University of Pennsylvania, USA

**16:35 - 16:45** **Discussion**

**Poster session II (Even numbered abstracts)**

**16:45-17:25**

**Sponsored Evening Seminar I** **< Boehringer Ingelheim >**

**Chair:** David Fiorentino and Joerg Wenzel

**17:30 - 18:00** 1. **The diagnosis and treatment of PM/DM-ILD**
Masatoshi Jinnin
Department of Dermatology, Faculty of Medicine, Wakayama Medical University, Wakayama, Japan

**18:00-18:30** 2. **Diagnosis, management and therapeutic strategy for interstitial lung disease associated with systemic sclerosis**
Yoshihide Asano
Department of Dermatology, Tohoku University School of Medicine, Japan

**Dinner (Buffet)**

**18:30 - 20:00**

# Timetable

**Day 2 (May 10, Wednesday)**

---

**8:15** **Registration**

**Sponsored Morning Seminar I** **< UCB Japan >**

**Chair:** Tamihiro Kawakami and Toshiyuki Yamamoto

**8:30 - 9:00** **1. Psoriasis and IL-17 family**

Rei Watanabe

Department of Integrative Medicine for Allergic and Immunological Diseases, Faculty of Medicine, Osaka University, Japan

**9:00 - 9:30** **2. Role of the IL-17 family of cytokines in dermatitis: Insights from mouse models**

Susumu Nakae

Graduate School of Integrated Sciences for Life, Hiroshima University, Japan

**Conference Overview, Poster Award Ceremony**

**9:35 - 9:45** Minoru Hasegawa

**Session I:** **Pathogenesis of Dermatomyositis**

**Chair:** Galen Foulke and Manabu Fujimoto

**9:45 - 10:00** **1. Murine models of idiopathic inflammatory myopathy including dermatomyositis**

Naoko Okiyama

Department of Dermatology, Graduate School of Medical and Dental Sciences, Tokyo Medical and Dental University, Japan

**10:00 - 10:15** **2. Novel insights into autoantibodies and cancer risk in DM**

David Fiorentino

Department of Dermatology, Stanford University School of Medicine, USA

**10:15 - 10:25** **3. Cellular misfolded protein/MHC class II complexes are possible autoantibody targets for autoimmune diseases**

Noriko Arase[1], Hisashi Arase[2,3], Manabu Fujimoto[1,4]

[1]Department of Dermatology, Osaka University, Japan; [2]Department of Immunochemistry, Research Institute for Microbial Diseases, Osaka University, Japan; [3]Immunochemistry, Immunology Frontier Research Center, Osaka University, Japan; [4]Cutaneous Immunology, Immunology Frontier Research Center, Osaka University, Japan

**10:25 - 10:35** **Discussion**

**10:35 - 10:50** **Poster View, Break**

# Timetable

**Session II: Clinical Insights of Dermatomyositis (30 min)**

**Chair:** David Fiorentino and Claudia Guenther

**10:50 - 11:00** **1. Autoantibodies and clinical phenotypes in dermatomyositis**
Manabu Fujimoto
Department of Dermatology, Osaka University, Japan

**11:00 - 11:10** **2. Disease presentations in dermatomyositis patients with skin of color**
Michelle Lee, BA[1], L. Steven Brown,[2] Richard D. Sontheimer,[3] Benjamin F. Chong[1]
[1]Department of Dermatology, University of Texas Southwestern Medical Center, Dallas, TX, USA; [2]Department of Health Systems Research, Parkland Health, Dallas, TX, USA; [3]Department of Dermatology, University of Utah Health, Salt Lake City, UT, USA

**11:10 - 11:20** **Discussion**

**Session III: Treatment of Dermatomyositis**

**Chair:** Noriki Fujimoto and Joerg Wenzel

**11:20 - 11:35** **1. Novel DM targets**
David Fiorentino
Department of Dermatology, Stanford University School of Medicine, USA

**11:35 - 11:50** **2. Safety and Efficacy of Lenabasum, an oral CBR2 Agonist, in Patients with Dermatomyositis: A Phase 3 Randomized, Double Blind, Placebo-Controlled Trial**
Victoria P. Werth*[1,2], Barbara White[3], Nancy Dgetluck[3], Kathleen Hally[3], Scott Constantine[3], Rohit Aggarwal[4], David Fiorentino[5], Ingrid E. Lundberg[6], Chester V Oddis[7]
[1] U of Pennsylvania, Dermatology, Philadelphia, USA, [2] Corporal Michael Crescenz VAMC, Philadelphia, USA, [3] Corbus Pharmaceuticals, Norwood, MA, USA, [4] U Pittsburgh, Dept of Rheumatology, Pittsburgh, USA, [5] Stanford U, Dermatology, Stanford University, USA, [8] Karolinska Institute, Division of Rheumatology, Dept of Medicine, Stockholm, Sweden

**11:50 - 12:00** **Discussion**

**12:00 - 12:10** **Break**

**Sponsored Luncheon Seminar II <Amgen>**

**Chair:** Benjamin Chong and Filippa Nyberg

**12:10 - 12:40** **1. Seeking precision medicine based on the pathophysiology of Behçet's disease**
Yohei Kirino
Department of Stem Cell and Immune Regulation, Yokohama City University Graduate School of Medicine, Japan

**12:40 - 13:10** **2. IFNα producing innate cells in systemic lupus erythematosus**
Sachiko Miyake [1,2], Taigai Kuga [1,2], Goh Murayama [1,2], Ken Yamaji[2], Naoto Tamura[2],

and Asako Chiba [1]
[1]Department of Immunology, Juntendo University Graduate School of Medicine, Japan
[2]Department of Rheumatology, Juntendo University Graduate School of Medicine, Japan

**13:10-13:20** **Break**

**Session IV:** **Rheum-Derm Grand Rounds**

**Chair:** Lauren Graham and Filippa Nyberg

**13:20 - 14:25**

**1 (CO1)** **Linear cutaneous lupus erythematosus – an important differential for blaschkoid dermatoses**

Ysabel Regina H. Ortiz, Juan Paolo David S. Villena, Eileen Liesl A. Cubillan
Department of Dermatology, University of the Philippines – Philippine General Hospital, Philippine

**2 (CO2)** **Improvement of systemic sclerosis-associated digital ulcers after UVA1 phototherapy**

Meagan Mandabach Olivet, Kevin Yang, Lauren V Graham
Department of Dermatology, The University of Alabama at Birmingham, USA

**3 (CO3)** **Paraneoplastic interface dermatitis presenting as exfoliative erythroderma**

Hana Ahmed, Lauren V Graham
Department of Dermatology, The University of Alabama at Birmingham, USA

**4 (CO4)** **ACLE in a young woman after Dapsone treatment**

Amanda Rödöö and Filippa Nyberg
Karolinska University Hospital. Stockholm, Sweden

**5 (CO5)** **Dermatomyositis with minimal myositis and MI2 autoantibodies**

Britta Krynitz and Filippa Nyberg
Department of Pathology and department of Dermatology, Karolinska University Hospital. Stockholm, Sweden

**6 (CO6)** **Cutaneous lupus erythematosus after Nivolumab treatment**

Jan Lapins and Filippa Nyberg
Department of Dermatology, Karolinska University Hospital. Stockholm, Sweden

**14:25-14:30** **Closing Remarks**

European Representative

**Peeling Off the Posters**

# ~ *Abstracts* ~

# Day 1

## Session I: Pathogenesis and Clinical Insights of Scleroderma

---

### 1. Outcomes research in morphea

Heidi Jacobe

Department of Dermatology, UT Southwestern Medical Center, USA

Morphea or localized scleroderma is a poorly studied disorder with inconsistent reports of clinical outcomes and frequency and type of associated extracutaneous manifestations. The Morphea in Adults and Children Cohort contains 900 participants followed annually by a single investigator. This work has allowed our team to better understand both cutaneous and extracutaneous outcomes in morphea. Key findings include better disease subtyping, understanding of clinical disease course, risk stratification for extracutaneous disease, and new patient reported outcomes.

# Day 1

## 2. The Role of B Cells in Systemic Sclerosis

Takemichi Fukasawa, Ayumi Yoshizaki, Asako Yoshizaki, Shinichi Sato

Department of Dermatology, The University of Tokyo, Japan

B cells have long been considered to be specialized for antibody production and to play no other important role in the immune system. Recently, however, it has become clear that B cells have various functions other than antibody production. For example, like macrophages and dendritic cells, B cells have antigen-presenting ability and produce a wide variety of cytokines that act directly on T cells and other immune cells, inducing their activation and differentiation. B cells are thought to play a central role in the immune system.

B cells have been shown to play a central role in autoimmune reactions and have been strongly suggested to play an important role in the pathogenesis of systemic sclerosis (SSc), an autoimmune disease. SSc is a chronic disease characterized by skin and visceral sclerosis. In SSc, immune abnormalities, fibrosis, and vasculopathy are known as the triad of symptoms, and B-cell abnormalities are thought to play a major role in the pathogenesis of the disease. Therefore, treatment targeting B cells is expected to be a new therapeutic strategy for SSc. In particular, B cell-depleting therapy using antibodies against CD20, which is specifically expressed on the surface of B cells, has been shown to be effective in various autoimmune diseases, further emphasizing the importance of B cells. Recently, an investigator-initiated clinical trial in patients with SSc demonstrated the efficacy of B-cell depletion therapy in the treatment of skin sclerosis and interstitial lung disease. This suggests that immune abnormalities, especially B cells, are deeply involved in the fibrosis of SSc.

In light of the above, in this presentation, we will discuss the diverse roles of B cells in the pathogenesis of SSc, including their ability to produce cytokines and their impact on other immune systems and their ability to produce antibodies.

# Day 1

## 3. Novel Comprehensive Detection Method for Autoantibodies and Its Clinical Application

Ayumi Yoshizaki

Department of Dermatology, University of Tokyo Graduate School of Medicine, Japan

In many systemic autoimmune diseases, autoantibodies appear in the serum before the manifestation of symptoms. This suggests that autoantibodies are closely involved in the pathogenesis and progression of autoimmune diseases. However, there is little evidence that autoantibodies themselves are directly pathogenic except in some organ-specific autoimmune diseases. Nevertheless, the type of autoantibodies detected in patients with systemic autoimmune diseases is associated with the manifestations, severity, and prognosis. Therefore, the identification of autoantibodies is useful not only for diagnosis, but also for determining therapeutic strategies.

Autoantibodies could theoretically exist for as many proteins as there are mRNAs to be translated from. However, transcriptome-wide autoantibodies have not been adequately investigated. Thus, the clinical value of autoantibodies remains to be fully elucidated. We therefore used a unique, comprehensive autoantigen array chip generated from a transcriptome-wide cDNA library to discover new clinical value of autoantibodies in systemic sclerosis, psoriasis, cutaneous arteritis, and even malignant melanoma.

In this presentation, I will outline the new value of autoantibodies revealed by our proprietary technology of transcriptome-wide comprehensive autoantibody assay. Furthermore, the autoantigen used in this technology is a protein that retains its three-dimensional structure, enabling the measurement of autoantibodies with high sensitivity and specificity. For systemic scleroderma and autoimmune myositis, 33 and 47 autoantibodies, respectively, can already be measured on a commercial basis, which is useful in clinical practice. A new autoantibody examination method for systemic sclerosis and autoimmune myositis, named A-Cube, will also be introduced in this presentation.

## Day 1

# Session II: Basic and Clinical Studies of Rheumatic Diseases

---

### 1. (RO-P1) Long-term efficacy of rituximab in patients with systemic sclerosis: follow-up of the DESIRES trial with a focus on immunoglobulin levels

Ai Kuzumi, Satoshi Ebata, Takemichi Fukasawa, Shinichi Sato, Ayumi Yoshizaki.

Department of Dermatology, Graduate School of Medicine, The University of Tokyo, Japan

Systemic sclerosis (SSc) is a progressive fibrosing disorder with poor prognosis. Although B cell depletion therapy with rituximab emerges as a promising therapeutic option for SSc, its long-term efficacy is still unclear. In addition, little information is available on potential response markers to evaluate and predict its efficacy in SSc. Here, we reported a long-term follow-up of 29 SSc patients who continued to receive rituximab (375 mg/$m^2$ once every week for four weeks) after completion of the double-blind, investigator-initiated, randomised, placebo-controlled (DESIRES) trial with detailed clinical and laboratory data. Significant improvements in modified Rodnan skin score (MRSS) and percentage of predicted forced vital capacity (FVC) were observed after one and three courses of rituximab, respectively, both of which were sustained after a median follow-up of 96 weeks. High responders (MRSS improvement of ≥ 9; n = 16) experienced a greater decrease in serum levels of IgG and IgA compared with low responders (MRSS improvement of ≤ 8; n = 13). In particular, the decrease in serum IgA levels significantly correlated with the improvement in MRSS. At the end of the follow-up, hypogammaglobulinemia (isolated low IgM [n = 7]; isolated low IgG [n = 1]; and isolated low IgA [n = 1]) was observed in nine patients and associated with greater improvement in FVC% predicted, especially in those with low IgM. In summary, rituximab significantly improved skin and lung fibrosis in SSc patients in a long-term follow-up. Given the progressive nature of the disease, this result is encouraging and supports the role of rituximab as a disease-modifying therapy for SSc. This study also highlights the clinical relevance of serum immunoglobulins, which might reflect the dynamics of B cells during rituximab therapy in SSc. Serum immunoglobulins may be a promising tool to evaluate and predict the efficacy of rituximab in SSc, and should be further explored as potential response markers

### 2. (RO-P2) Updates on the current state of management and

# Day 1

## assessment of the highlighted risk for atherosclerotic cardiovascular events in an established cohort of patients with lupus erythematosus: a multifactorial issue

Megan Zhao, BA[1,2], Rui Feng, PhD[3], Kevin Jon Williams, MD[4*], Victoria P. Werth, MD[1,2*]

[1]Corporal Michael J. Crescenz Veterans' Administration Medical Center, Philadelphia, PA, USA; [2]Department of Dermatology, University of Pennsylvania, Perelman School of Medicine, Philadelphia, PA; [3]Department of Biostatistics, University of Pennsylvania, Perelman School of Medicine, Philadelphia, PA; [4]Department of Cardiovascular Sciences, Department of Medicine, Lewis Katz School of Medicine at Temple University, Philadelphia, PA, USA. *Co-senior authors

Patients with lupus erythematosus (LE) are at a heightened risk for clinical events, chiefly heart attacks and strokes, caused by atherosclerotic cardiovascular disease (ASCVD). To address this problem, we recently proposed new guidelines for categorization of levels of risk for future ASCVD events specifically in LE patients, with corresponding recommendations for management. We included all participants in our established UPenn Longitudinal Lupus Cohort of patients with cutaneous LE, without or with concurrent systemic LE (n=370, LE-ASCVD Study Cohort, years 2007 to 2021). Of our LE-ASCVD Study Cohort, 336/370 (90.8%) had a designated primary care physician. By the new guidelines, the most recent plasma low-density lipoprotein cholesterol (LDLc) levels were above goal for 252/370 (68.1%) of the LE-ASCVD Study Cohort. Of the Study Cohort, 266 (71.9%) had hypertension, which was under- or un-treated in 198/266 (74.4%). Of current smokers, 51/63 (81.0%) have no chart documentation of smoking cessation counseling or clinic referral. Diabetes was generally well-managed, and hypertriglyceridemia was uncommon. Of the LE-ASCVD Study Cohort, 254 patients qualified for two widely used online calculators that estimate the risk of an ASCVD event in the next 10 years ("10-year ASCVD event risk"): the ACC-ASCVD Risk Estimator Plus and QRisk3. We also stratified these 254 patients into the categories of ASCVD event risk defined by Keyes et al. Surprisingly, these three methods for estimating ASCVD event risk showed clinically meaningful agreement for only 100/254 (39.4%), i.e., discordance for over 60% of LE patients that could affect their clinical management. The actual documented rate of ASCVD events for patients in the first 10 years after enrollment into the Cohort was 22.3% (95% CI 16.9%, 27.4%), indicating a high-risk population despite a preponderance of women and a median age of only 47 years at enrollment. We conclude that cutaneous LE patients are under-treated compared with the new guidelines and, accordingly, these patients experience a substantial burden of major adverse ASCVD events. Moreover, it is unclear how to accurately assess future ASCVD event risk in cutaneous LE patients – except that it is high – and this uncertainty may complicate clinical management. Efforts are underway to improve ASCVD event risk estimation and guideline implementation in lupus patients.

# Day 1

## 3. (RO-P3) Lack of cutaneous B cells differentiates lupus-like disease in MRL/LPR mice from human discoid lupus

Tim Curran[1], Joshuah Yon[2], Christopher T. Richardson[1]

1. Dermatology, University of Rochester Medical Center, Rochester, NY, United States
2. University of Rochester, Rochester, NY, United States

Two recent studies have shown that a prominent B cell signature differentiates discoid lupus from other cutaneous lupus subtypes. The MRL/lpr mouse model of lupus is one of the most studied and one of the few to spontaneously develop significant cutaneous disease, which can be induced earlier with ultraviolet B (UVB) radiation. In this model, systemic disease is B cell dependent and some cutaneous features resemble discoid lupus. We sought to investigate whether MRL/lpr skin lesions also exhibit a prominent B cell signature. The dorsoscapular skin of 4-week-old MRL/lpr mice (n=8) was exposed to UVB for 6 weeks to induce skin lesions. A second group (n=8) was unexposed to UVB. Skin lesions developed to a variable extent in both groups by 14 weeks of age (6/8 mice with UVB, 4/8 mice without UVB), at which point biopsies were taken from the dorsoscapular skin of all 16 mice. Immunohistochemistry revealed a significant CD3+ T cell infiltrate in all skin biopsies. No CD20+ B cells were observed in any skin biopsies. These results were confirmed by quantitative RT-PCR analysis, which showed significant CD8, CD4, and MX1 expression consistent with a T cell infiltrate and interferon signature, but no expression of CD20 in MRL/lpr skin. Expression of CD4, CD8, and CD20 was robust in the spleen of the same mice. The complete lack of B cells in MRL/lpr skin lesions indicates that cutaneous disease in MRL/lpr mice is an inadequate model for human discoid lupus, but may better resemble other cutaneous lupus subtypes such as subacute cutaneous lupus.

# Day 1

## 4. (RO-P4) Multiplexed mass cytometry of cutaneous lupus erythematosus and dermatomyositis skin

Mariko Ogawa-Momohara[1,2,3] Thomas Vazquez[1,2], Meena Sharma[1,2], Joshua Dan[1,2], Grant Sprow[1,2], Victoria P. Werth[1,2]

[1]Corporal Michael J. Crescenz VAMC, Phil, PA, 19104; [2] Dermatology, University of Pennsylvania, Phil, PA, USA; [3] Dermatology, Nagoya University Graduate School of Medicine, Nagoya, Japan.

Cutaneous lupus erythematosus (CLE) and dermatomyositis (DM) are both characterized histologically by interface dermatitis. Although some studies suggest that anti-CD20 treatment is effective for the cutaneous manifestations of CLE and DM, responses differ between them. We therefore sought to characterize the cell compartment in CLE and DM skin to better understand the cutaneous immunopathogenesis of these autoimmune connective tissue diseases. We recruited patients from our prospective CLE and DM databases at the University of Pennsylvania with Institutional Review Board approval. Age-matched healthy controls (HC) were selected from the Penn Skin Biology and Disease Resource Center. We performed imaging mass cytometry on 43 archived, lesional skin biopsies (11 DM, 5 ACLE, 7 DLE, 10 SCLE and 10 HC) using 28 distinct cell markers, cytokines, and immunoglobulins to identify the immunophenotype in each group. We found 11 significant lymphocyte cell groups among all samples. Cell counts of T cells/region of interest (ROI) ($CD4^+$, $CD8^+$, Treg and $CD45RA^{+\backslash}$) were most upregulated in DLE compared with HC ($p<0.01$). $CD20^+$ cells were increased in DM compared to HC ($p<0.001$). $CD38^+$ B cell counts/ROI were significantly increased in DM and DLE compared to HC ($p<0.01$). Conventional DCs were increased in DM compared to HC ($p<0.001$). IL10 was highly expressed by plasma cells relative to other cell types. We identified several differences in the B and T lymphocyte compartments in CLE and DM. DLE skin demonstrated increased $CD45RA^+$ T cells and $CD38^+$B cell, whereas $CD20^+$ B cell and cDCs were increased in DM. The primary IL10 producing cell in DM CLE and HC was the plasma cell in the skin.

# Day 1

## 5. (RO-P5) The Total Improvement Score (TIS) in a Phase 3 Clinical Trial for Dermatomyositis (DM): Room for Improvement?

J Dan[1,2], D Lim[1,2], R Pandya,[1,2] J Concha[1,2], J Kleitsch,[1,2] G Sprow[1,2], N Kodali[1,2], D Diaz[1,2], B White[3], VP Werth[1,2]

[1]Dermatology, PSOM, UPenn, Phil, PA, USA; [2]CMC VAMC, Phil, PA, USA; [3]Corbus Pharmaceuticals, Norwood, MA, USA

TIS is a composite score proposed as a primary efficacy measure in DM clinical trials. We hypothesize that TIS may not adequately capture skin improvement in DM, as it does not include any direct measure of skin activity and may use redundant components. We also hypothesize that a new composite outcome, named Dermatomyositis Outcomes for Muscle and Skin (DMOMS), which includes components of TIS and a skin-specific outcome, is better suited to capture improvements in DM disease activity. DMOMS includes improvement from baseline in Manual Muscle Test (MMT), Physician's Global Assessment (PGA), and Patient's Global Assessment (PtGA), all scored as in TIS except a 50% increase in weight for PtGA. It also includes the Cutaneous Dermatomyositis Disease Area and Severity Index-Activity (CDASI-A) score, weighted the same as MMT.

To compare efficacy of scores, data from a Phase 3 trial of lenabasum in DM were evaluated (Baseline vs Week 52). Pearson's correlation assessed for redundancy ($r>0.8$). PGA and Extramuscular Global Assessment in TIS score were redundant ($r=0.827$). Subjects with a 10-point improvement in MMT or 11-point improvement in CDASI-A score at Week 52 (N=96) were considered responders (R). Mean (SD) TIS and DMOMS scores were compared for R and non-responders (NR) using Student's t-test. CDASI-A R (n=36) vs NR (n=60) had mean TIS of 47 (13) vs 30 (18), $p<0.001$ and DMOMS of 64 (16) vs 29 (22), $p<0.001$. MMT R (n=27) vs NR (n=69) had a mean TIS of 49 (15) vs 31 (17), $p<0.001$ and DMOMS of 66 (18) vs 32 (23), $p<0.001$. Compared to TIS, DMOMS is simpler composite score without redundant components (4 components vs 6). It provides about twice the treatment effect for R vs NR in both muscle and skin without any increase in score in NR and assigns greater weight to PtGA. DMOMS may be better suited to detect treatment effect in DM clinical trials that include patients with all DM phenotypes, even with a smaller sample size.

# Day 1

## Sponsored Luncheon Seminar I

Janssen Pharma

---

### 1. Treatment management for digital ulcers in systemic sclerosis ~Role of bosentan in the treatment of DUs in SSc~

Sei-ichiro Motegi

Department of Dermatology, Gunma University, Japan

Systemic sclerosis (SSc) is an autoimmune connective tissue disorder characterized by the development of fibrosis in the skin and internal organs as well as by vascular dysfunction, Vascular disorders in SSc appear early in the course of the disease and present with a variety of symptoms, including Raynaud's phenomenon, abnormal capillaries in nailfold, telangiectasia, digital pitting scars, digital ulcers, and gangrene. Vascular lesions are also associated with the pathogenesis of pulmonary arterial hypertension and renal crisis.

The fingers and toes are prone to ulceration because they are susceptible to cold and physical trauma, as well as to circulatory disturbance due to skin fibrosis and abnormal vascular function, resulting in decreased peripheral blood flow. Digital ulcers associated with SSc are often difficult to treat, and bacterial infection can worsen the ulcers, resulting in osteomyelitis or arthritis, and sometimes leading to amputation of the digits. Therefore, early and appropriate treatment is essential. This presentation will discuss treatment strategies for digital ulcers associated with SSc. In particular, the mechanism of action of endothelin receptor antagonist (bosentan), suitable patients for its use, actual methods of use, and points to be considered will be introduced. We will also present the therapeutic efficacy and safety of cases treated with bosentan at our institution.

# Day 1

## 2. Diagnosis and treatment for systemic sclerosis-associated pulmonary arterial hypertension

Takashi Matsushita

Department of Dermatology, Kanazawa University, Japan

Systemic sclerosis (SSc) is a connective tissue disease characterized by fibrosis and vascular lesions of the skin, lungs, and other internal organs with a background of autoimmune phenomena represented by antinuclear antibodies. SSc patients require early treatment because tissue damage gradually accumulates, leading to severe multiorgan damage. Until now, it has been challenging to diagnose early-stage cases without skin sclerosis, but the ACR/EULAR classification criteria were published in 2013, making it possible to make an early diagnosis of SSc based on other skin symptoms and the presence of disease-specific antibodies, even in the absence of skin sclerosis. Interstitial lung disease is the leading cause of death in SSc (35.2%), and pulmonary arterial hypertension (PAH) is the second leading cause of death in SSc (25.8%). Compared to PAH associated with other connective tissue diseases, the prognosis is inferior, with a reported 5-year survival rate of 43% and a 10-year survival rate of 21%. Therefore, early diagnosis of SSc-PAH is essential. Furthermore, early diagnosis of SSc-PAH by screening with echocardiography has been shown to improve prognosis. Consequently, it is important to perform annual echocardiographic screening to detect PAH early and intervene in its treatment. PAH drugs are classified into three systems: prostacyclins, NO, and endothelins, all of which exert their therapeutic effects by dilating pulmonary blood vessels. One drug from each system is selected for combination therapy, and up to three drugs can be used in combination. It has been reported that treatment results are better when multiple agents are used than when a single agent is used. Furthermore, PAH drugs have been shown to improve the prognosis of SSc-PAH. This talk will outline the importance of early diagnosis and treatment of SSc-PAH.

## Day 1

# Session III: Pathogenesis of CLE

---

## 1. Overview

Joerg Wenzel

Department of Dermatology and Allergy, University Hospital Bonn, Bonn, Germany

# Day 1

## 2. (Immuno-) histological stratification of CLE patient for targeted therapies

Joerg Wenzel

Department of Dermatology and Allergy, University Hospital Bonn, Bonn, Germany

# Day 1

## 3. Tape Stripping: A novel, non-invasive method for identifying and measuring cutaneous lupus activity

Joseph F. Merola, MD MMSc

Harvard Medical School, Brigham and Women's Hospital, Boston, MA, USA

Tape stripping has been validated initially as a method for risk stratifying melanocytic neoplasms and differentiating melanoma from dysplastic nevi. This work has led to a commercially available test and offers a non-invasive option in the appropriate clinical scenarios. Our group was interested in the opportunity to consider non-invasive methods of measuring cutaneous lupus erythematosus (CLE) activity utilizing this method in a proof of concept study. We demonstrated that non-invasive tape sampling represented an viable alternative to serial evaluation of CLE lesions. The technique evaluated the ability to collect mRNA from the skin surface of participants with CLE and healthy volunteers. 94 Candidate immune genes were amplified via mRNA collected using the tape device and an IFN-dominant gene cluster differentiated CLE from healthy skin as well as CLE-unaffected skin. Two clusters, an IFN-dominant and CLE-associated cluster, were demonstrated and correlated well with punch biopsy specimens. Since that time, other groups have evaluated tape stripping in a variety of inflammatory cutaneous dermatoses including atopic dermatitis and psoriasis in larger cohorts which yielded RNA-seq tape strip profiling able to detect distinct immune and barrier signatures in lesional and non-lesional atopic and psoriasis skin.

Merola JF, Wang W, Wager CG, Hamann S, Zhang X, Thai A, Roberts C, Lam C, Musselli C, Marsh G, Rabah D, Barbey C, Franchimont N, Reynolds TL. RNA tape sampling in cutaneous lupus erythematosus discriminates affected from unaffected and healthy volunteer skin. Lupus Sci Med. 2021 Mar;8(1):e000428. doi: 10.1136/lupus-2020-000428. PMID: 33658303; PMCID: PMC7931768.

## Day 1

# Session IV: Special Lecture

---

## Identification of pathological pathways of SLE from the immune cell transcriptome

Keishi Fujio

Department of Allergy and Rheumatology, Graduate School of Medicine, The University of Tokyo, Japan

Prognosis of autoimmune diseases is improving significantly due to advances in molecular targeted therapy. However, even molecular-targeted therapies have been found to be ineffective in some cases. It is essential to elucidate the pathophysiology of autoimmune diseases, such as the immunological pathways responsible for the disease, in order to provide better treatment. Recent advances in technology enabled the development of comprehensive analysis of immune cells, and we are conducting functional genomics research to analyze the genome's effect on the transcriptome in the immune cells of autoimmune diseases including SLE. In Japanese patients with SLE, analysis of expression quantitative trait locus (eQTL) effects has revealed that B cells are the immune cells most associated with the genetic risk. In addition, transcriptome analysis of SLE revealed that the mitochondrial and complement pathways are the Disease-state signatures that are upregulated in clinically stable SLE, and that the cell cycle and ribosomal pathways are the Disease-activity signatures that are upregulated in active SLE. Interestingly, mycophenolate mofetil (MMF), an immunosuppressive drug effective in SLE, inhibited the cell cycle pathway in the Disease-activity signature. In addition, enhancement of the mitochondrial pathway in the Disease-state signature was associated with organ damage in SLE, suggesting a link between residual pathways and prognosis in apparently stable SLE. Furthermore, when we examined the association with susceptibility genes for SLE, we found that a Disease-state signature rather than a Disease-activity signature was associated, suggesting that previous genome-wide association analyses may not have captured the pathways associated with disease activity. These results indicate that analysis of the transcriptome of immune-competent cells in autoimmune diseases can identify pathways associated with disease activity and prognosis. Although much remains to be elucidated before clinical application, it is hoped that such an integrative approach will lead to a better understanding of pathological pathways in autoimmune diseases.

# Day 1

## Session V: Clinical Insights of CLE

---

### 1. The relationship between systemic lupus erythematosus and cutaneous lupus erythematosus

Benjamin F. Chong, MD, MSCS

Department of Dermatology, University of Texas Southwestern Medical Center, Dallas, TX, USA

Diagnosing systemic lupus erythematosus (SLE) in cutaneous lupus erythematosus (CLE) patients has implications for prognosis and treatment. The eleven American College of Rheumatology (ACR) SLE criteria have been used by practitioners to diagnosis SLE. Criticisms of these criteria include that they do not differentiate other skin diseases, and that they are heavily weighted towards cutaneous manifestations. The 2012 Systemic Lupus International Collaborating Clinics (SLICC) criteria and the 2019 European League Against Rheumatism (EULAR)/ACR recommended new SLE diagnostic criteria to address these specific limitations, amongst others. Large epidemiological studies in Minnesota and Sweden have noted that close to 20% of CLE patients may develop SLE. However, our group has found differences in rates of progression from CLE to SLE in our patient cohort using the ACR, SLICC, and EULAR/ACR criteria. Furthermore, our group and others have indicated various clinical and laboratory indicators, such as widespread discoid lesions, arthralgias/arthritis, high titers of anti-nuclear antibodies (ANAs) and higher number of baseline criteria have been identified as risk factors for SLE development. To assess the risk of SLE development in CLE patients, complete skin exams, joint assessments, and laboratory tests including ANA should be performed regularly. Prospective studies following CLE patients who do or do not develop SLE are still ongoing at University of Texas Southwestern Medical Center to identify biomarkers that improve our assessment of risk of systemic spread in these patients.

# Day 1

## 2. Outcome measures in cutaneous lupus studies

Joseph F. Merola, MD MMSc

Harvard Medical School, Brigham and Women's Hospital, Boston, MA, USA

There have been numerous barriers to the regulatory approval of new investigational products, specifically for cutaneous lupus erythematosus (CLE) as an indication, in the US. Further, a lack of consensus among researchers has led to problematic heterogeneity in data within observational and interventional studies in CLE, further compounding challenges to align datasets. While the CLASI represents the most well-known and validated measure to date, there remain hurdles on the regulatory acceptance of the CLASI and its derivatives in the US, where other authorities have been more accepting. To that end, we have developed a 'working core outcome set for cutaneous lupus erythematosus' to provide a practical approach to an urgent unmet need, aligning our research community around both clinical measures and patient reported measures to be used presently. To respond to a consistent US regulatory request, we have also developed the CLA-IGA-R (Cutaneous Lupus Activitity Investigator Global Assessment – Revised), an IGA based tool to measure CLE disease activity, and meant to complement existing assessor measures, namely the CLASI. The instrument is now being used as primary, secondary and exploratory endpoints in multiple phase 2/3 trials and registries.

Guo LN, Perez-Chada LM, Borucki R, Nambudiri VE, Werth VP, Merola JF. Development of a working core outcome set for cutaneous lupus erythematosus: a practical approach to an urgent unmet need. Lupus Sci Med. 2021 Dec;8(1):e000529. doi: 10.1136/lupus-2021-000529. PMID: 34969875; PMCID: PMC8718411.

# Day 1

## 3. Heterogeneity of response to antimalarials and type I interferons related to dendritic cells

Victoria P. Werth[1,2], DeAnna Diaz[1,2], Felix Chin[1,2], Meena Sharma[1,2], Thomas Vazquez[1,2], Jay Patel[1,2]

[1]Dermatology, CMCVAMC and [2]Department of Dermatology, University of Pennsylvania, USA

The pathogenesis of cutaneous lupus erythematous (CLE) is multifactorial and CLE is difficult to treat due to heterogeneity of inflammatory processes between patients. Antimalarials such as hydroxychloroquine (HCQ) and quinacrine (QC) have long been first-line systemic therapy; however, many patients do not respond and require systemic immunosuppressants with undesirable side effects. Given the complexity and unpredictable responses in CLE, we sought to identify the immunologic landscape of CLE patients stratified by subsequent treatment outcomes to identify potential biomarkers of inducible response. We performed imaging mass cytometry with 48 treatment-naïve skin biopsies of HCQ responders, QC responders, and non-responders (NR) to analyze multiple immune cell types and inflammatory markers in their native environment in CLE skin. Patients were stratified according to their subsequent response to antimalarials to identify baseline immunophenotypes which may predict response to therapy. HCQ responders demonstrated increased CD4 T cells compared to QC. NR had decreased Tregs compared to QC and increased central memory T cells compared to HCQ. QC responders expressed increased phosphorylated (p) STING and IFNκ compared to HCQ. pSTING and IFNκ localized to conventional dendritic cells and positively correlated on a tissue and cellular level. Neighborhood analysis revealed decreased regulatory cell interactions in NR patients. Hierarchical clustering revealed NR groups separated based on pSTAT2/3/4/5, pIRF3, Granzyme B, pJAK2, IL4, IL17, and IFNγ. Plasmacytoid dendritic cells (pDCs) did not stain for type 1 interferon (IFN-1), but were positive for granzyme B (GZMB) on imaging mass cytometry. Skin-eluted and circulating pDCs from CLE subjects expressed significantly less IFNα than healthy control pDCs upon toll like receptor (TLR) 7 stimulation *ex vivo* ($p<0.0001$). These findings demonstrate differential immune compositions between CLE patients, guiding the future for precision-based medicine and treatment response.

# Day 1

## Session VI: Treatment of CLE

### 1. Recent findings about antimalarial in cutaneous lupus erythematosus

Francois Chasset

Sorbonne Université, Faculté de médecine, Service de dermatologie et allergologie, Hôpital Tenon, AP-HP, Paris F-75020, France

Antimalarials, particularly hydroxychloroquine (HCQ) and chloroquine (CQ), are the cornerstone of the treatment of both systemic lupus erythematosus (SLE) and cutaneous lupus erythematosus (CLE). HCQ and CQ are recommended as first-line oral agents in all the CLE guidelines. Because of its potential therapeutic action in covid-19, the number of publications regarding HCQ has considerably increased in the last three years, highlighting the potential severe toxicity. In this presentation, we will review recent advances in the efficacy of antimalarials in CLE with a focus on the therapeutic strategy: switching from HCQ to CQ, adding quinacrine, and increasing HCQ dose based on blood concentration. We will discuss the possibility of decreasing the dose of HCQ or stopping HCQ in cases of CLE remission. The long-term use of HCQ is associated with potentially severe retinal toxicity. Recent ophthalmological studies have reported a strong association between the daily dose of HCQ and the risk of retinopathy. A cut-off of 5 mg/kg/day has been suggested as a safe dose and has been implemented in the recent CLE and SLE guidelines. However, recent studies have suggested that this dose is associated with an increase in moderate-to-severe SLE flares. We will review these studies to identify the potential optimal dose of HCQ with a focus on the difference between doses prescribed and delivered by the pharmacist.

# Day 1

## 2. Refractory cutaneous lupus: Use of thalidomide, lenalidomide and lenalidomide for refractory lupus skin disease.

Francois Chasset

Sorbonne Université, Faculté de médecine, Service de dermatologie et allergologie, Hôpital Tenon, AP-HP, Paris F-75020, France

Cutaneous lupus erythematosus (CLE) may cause extensive skin damage leading to aesthetic prejudice and poor quality of life. Antimalarial agents are recommended as first-line systemic therapy for CLE patients with moderate to severe skin lesions. In case of failure of antimalarial agents, a European expert consensus for CLE treatment and EULAR recommendation for SLE recommend adding quinacrine, methotrexate, retinoids, dapsone or mycophenolate mofetil. However, the overall efficacy of these second-line treatments is approximately 50% depending on CLE subtypes and may be associated with potential toxicity. Thalidomide (a-N-phthalimidoglutarimide) is currently recommended as a “rescue” therapy in patients with severe and refractory CLE treatment. However, in France, a Temporary Recommendations for Use has been granted for thalidomide in CLE after failure of antimalarials as second-line agent. Moreover, in a systematic literature review including patients the pooled rate of response to thalidomide 50-100mg/day was 90% (95% CI, 85-94), with similar response rates between CLE subtypes. The clinical benefits need to be balanced against potential adverse events including high teratogenicity, peripheral neuropathy, thromboembolic events. In case of failure of thalidomide, lenalidomide 5mg/day a 4-amino-glutamyl analogue of thalidomide has shown promising results with partial response of 88% in a retrospective study of 40 patients. At the molecular level, thalidomide and lenalidomide bind cereblon, a protein which belong to a molecular complex which increase ubiquitination and degradation of IKZF1 (Ikaros) and IKZF3 (Aiolos). More recently, iberdomide, a specific cereblon Immunomodulator was developed with promising results in SLE. We will discuss recent findings and our clinical experience in the use of these drugs in CLE patients

# Day 1

## 3. Update on clinical trials for cutaneous lupus erythematosus

Victoria P. Werth[1,2]

[1]Dermatology, CMCVAMC, [2]Department of Dermatology, University of Pennsylvania, USA

Cutaneous lupus erythematosus (CLE) is an autoimmune disease that can occur with or without underlying systemic lupus erythematosus (SLE) and often has a profoundly negative impact on patient quality of life. There is substantial need for new and more effective therapies to treat CLE. CLE has a multifactorial pathogenesis that involves several key immune cells and pathways, including abnormalities in innate (e.g., type 1 interferon pathways) and adaptive immune responses (e.g., B and T cell autoreactivity), presenting multiple opportunities for more targeted therapies that do not require immunosuppression. Here we review several emerging therapies and their efficacy in CLE. Anifrolumab and belimumab have both been approved for the treatment of SLE in recent years, and clinical trial evidence suggests some forms of CLE may improve with these agents. Promising new therapies in development that are being evaluated with CLE-specific outcome measures include litifilimab and daxdilimab, which target plasmacytoid dendritic cells (pDCs), iberdomide, a cereblon modulator, and deucravacitinib, a TYK2 inhibitor. These novel therapies all have demonstrated clinical benefit in some forms of CLE in phase 1-2 trials. Other therapies which target molecules believed to play a role in CLE pathogenesis, such as janus kinases (JAKs), spleen tyrosine kinase (SYK), interferon γ (IFNγ), IL-12, and IL-23, have been evaluated in lupus clinical trials with skin-specific outcomes but failed to meet their primary endpoints.

## Day 1

# Sponsored Evening Seminar I

Boehringer Ingelheim

---

### 1. The diagnosis and treatment of PM/DM-ILD

Masatoshi Jinnin

Department of Dermatology, Faculty of Medicine, Wakayama Medical University, Wakayama, Japan

The disease concept of polymyositis/dermatomyositis-associated interstitial lung disease (PM/DM-ILDs) is thought to be included in connective tissue disease-associated ILD (CTD-ILD) or idiopathic inflammatory myopathy-associated (IIM-ILD).
Among the various ILDs, nintedanib is available for progressive fibrosing ILD (PF-ILD). Some of the CTD-ILDs or some of the PM/DM-ILDs can be regarded as PF-ILD and can be treated with nintedanib. PM/DM-ILDs, like other CTD-ILDs, are characterized by both inflammatory and fibrotic properties. At present, treatment option is focused on inflammation, but it is likely that a certain number of patients will require antifibrotic therapy. This talk will discuss the recent advances regarding the diagnosis of PM/DM-ILD and its treatment with nintedanib.

# Day 1

## 2. Diagnosis, management and therapeutic strategy for interstitial lung disease associated with systemic sclerosis

Yoshihide Asano

Department of Dermatology, Tohoku University School of Medicine, Japan

Systemic sclerosis (SSc) is a multisystem autoimmune disease characterized by vasculopathy and extensive fibrosis of the skin and various internal organs. Among a variety of cutaneous and visceral complications, interstitial lung disease (ILD) is the leading cause of mortality, accounting for approximately 35% of disease-related death in patients with SSc. In the recent decade, the management of SSc-ILD has been largely changed due to the results of key randomized controlled trials that have demonstrated the favorable disease-modifying effects of molecular-targeting therapies on this complication. Currently, nintedanib, an intracellular tyrosine kinase inhibitor with antifibrotic properties, has been widely approved in numerous countries against the fibrotic lung disease including SSc-ILD, while tocilizumab, an antibody against IL-6 receptor, and rituximab, an antibody against CD20, have been approved for SSc-ILD by the U.S. Food and Drug Administration and for SSc by the Pharmaceuticals and Medicals Devices Agency of Japan, respectively. These recent approvals challenge the management of SSc-associated fibrotic manifestations, including ILD. Under this situation, dermatologists are in an important, sentinel-like position to detect and respond to early indications of ILD in patients with SSc. In my talk, I will review the body of research on SSc-ILD and propose a practical approach for diagnosis, stratification, management, and therapeutic decision-making in this clinical context.

# Day 2

## Sponsored Morning Seminar I

UCB Japan

---

### 1. Psoriasis and IL-17 family

Rei Watanabe

Department of Integrative Medicine for Allergic and Immunological Diseases, Faculty of Medicine, Osaka University, Japan

Psoriasis is a T17 disorder characterized by IL-23/IL-17 signaling cascades. While IL-17A is regarded as a main player, other IL-17 family members are also possibly involved in the psoriatic pathogenesis. In particular, IL-17F has the highest homology with IL-17A. IL-17F is often secreted from IL-17A-producing cells in response with IL-23 stimulation. IL-17A and F share the same receptors and their functional activities are overlapped. On the other hand, their independent functionalities are also reported.

The biologics targeting both IL-17A and F is established as an effective treatment modality in psoriasis. At the same time, the typical treatment course suggests the distinct functional mechanism of the IL-17A/F inhibitor compared to the IL-17A-specific inhibitors.

In this seminar, I would like to think over the features of targeting both IL-17A and F in psoriasis treatment by overviewing the common and distinct involvement of these cytokines in the disease pathogenesis.

## Day 2

### 2. Role of the IL-17 family of cytokines in dermatitis: Insights from mouse models

Susumu Nakae

Graduate School of Integrated Sciences for Life, Hiroshima University, Japan

IL-17 has six family members (IL-17A, IL-17B, IL-17C, IL-17D, IL-17E (IL-25) and IL-17F). *Il17a* and *Il17f* genes are closely located on the same chromosome, and IL-17A and IL-17F, which have high homology in amino acid sequences, bind to the same receptors. On the other hand, IL-17A and IL-17F have distinct functions in certain settings including dermatitis. In addition, it has been reported that IL-17C and IL-25 are also involved in the pathogenesis of dermatitis. In my presentation, I will review the role of the IL-17 family of cytokines in dermatitis from the study using mouse model.

# Day 2

## Session I: Pathogenesis of Dermatomyositis

---

### 1. Murine models of idiopathic inflammatory myopathy including dermatomyositis

Naoko Okiyama

Department of Dermatology, Graduate School of Medical and Dental Sciences, Tokyo Medical and Dental University, Japan

Idiopathic inflammatory myopathies (IIMs) are characterized by inflammation of muscles and other organs, including interstitial lung disease (ILD). Dermatomyositis (DM) is an IIM with specific cutaneous manifestations. Several myositis-specific autoantibodies (MSAs) have been identified in IIMs and were found to be associated with distinct clinical features. Although MSAs are valuable for the diagnosis of IIMs, the pathogenic roles of these antibodies remain unknown.

To investigate the pathogenesis of IIMs, several animal models of experimental myositis have been established. Classical murine models of autoimmune myositis, experimental autoimmune myositis, and C protein-induced myositis are established by immunization with muscle-specific antigens, myosin, and skeletal C protein, respectively. A murine model of experimental myositis was generated by immunization with a murine recombinant histidyl-tRNA synthetase, Jo-1, in which muscle and lung inflammation reflecting anti-synthetase syndrome are induced, however, the development of this disease was not depending on anti-Jo-1 autoimmunity. On the other hand, the transfer of human IgGs from patients of another type of IIMs, immune-mediated necrotizing myopathy with anti-signal recognition particles and anti-3-hydroxy-3-methylglutaryl coenzyme A reductase antibodies, was found to induce complement-mediated myositis in recipient mice.

Furthermore, we established a murine model of autoimmune myositis induced by immunization of recombinant human transcriptional intermediary factor 1γ (TIF1γ), an autoantigen for MSAs. TIF1γ-induced myositis is mediated by TIF1γ-specific $CD8^+$ T cells, but not antibodies and B cells. We also have established another murine model of ILD induced by immunization of recombinant murine melanoma differentiation-associated gene 5 (MDA5), an autoantigen for MSAs. MDA5-induced ILD is mediated by MDA5-specific $CD4^+$ T cells, in which IL-6 has been raised as a potential therapeutic target. These new murine models reflecting MSA-related DM are useful tools for accurately understanding the pathological mechanisms underlying DM.

# Day 2

## 2. Novel insights into autoantibodies and cancer risk in DM

David Fiorentino, MD PhD

Department of Dermatology, Stanford University School of Medicine, CA, USA

Dermatomyositis (DM) is characterized by an increased risk of emergence of internal malignancy, with the highest cancer risk around the time of DM onset. Antibodies targeted against transcriptional intermediary factor 1-gamma (TIF1-γ)/TRIM33 are associated with a higher risk of cancer in adults with DM, especially within a 1-2 year window around disease onset. Despite this, many adult patients with DM and anti-TIF1-γ autoantibodies are never diagnosed with internal malignancy. Recent data suggest the autoantibody responses in these cancer-negative patients are strikingly more diverse than those DM patient in which cancer emerges. It is conceivable that some of these antigen-specific immune responses provide protection from the clinical emergence of nascent cancers associated with DM onset. These patient sera can provide a source for identification of autoantigens that are associated with cancer protection. Strikingly, some of these autoantibody targets are broadly shared across DM patients. CCAR1 (cell division cycle and apotosis regulator 1) is one of these targets. In two large U.S. cohorts, anti-CCAR1 antibodies are associated with cancer protection in DM patients to a level statistically indistinguishable from the general population. Several other autoantigens have been identified in a similar manner, with preliminary data suggesting that a similar phenomenon occurs in other (non anti-TIF1-γ) DM antibody subgroups. Altogether these findings have direct implications for cancer risk stratification that may help guide malignancy screening, and additionally provide evidence for a model in which cancers may be more commonly associated with rheumatic diseases than can be observed simply by observing those with clinical cancer emergence.

# Day 2

## 3. Cellular misfolded protein/MHC class II complexes are possible autoantibody targets for autoimmune diseases

Noriko Arase[1], Hisashi Arase[2,3], Manabu Fujimoto[1,4]

[1]Department of Dermatology, Osaka University, Japan
[2]Department of Immunochemistry, Research Institute for Microbial Diseases, Osaka University, Japan
[3]Immunochemistry, Immunology Frontier Research Center, Osaka University, Japan
[4]Cutaneous Immunology, Immunology Frontier Research Center, Osaka University, Japan

MHC class II molecules on antigen-presenting cells present foreign peptides to CD4-positive T cells. On the other hand, MHC class II loci are the genes most strongly associated with susceptibility to many autoimmune diseases. Our group found that misfolded proteins produced in the endoplasmic reticulum are transported to the cell surface as misfolded proteins/MHC class II complexes. These misfolded protein/MHC class II complexes have novel antigenic properties different from those of normal self-antigens, escape self-tolerance, and become targets for autoantibodies. Such mechanism has been demonstrated in rheumatoid arthritis, antiphospholipid syndrome, microscopic polyangiitis, and systemic lupus erythematosus.

SS-A/Ro52 is one of the target antigens of dermatomyositis. Ro52 molecule is transported to the cell surface by forming the Ro52/IgG/HLA-DR complex and this complex is found to be a target of autoantibodies in patients with anti-MDA5 antibody-positive dermatomyositis and in those with anti-synthetase syndrome. In the patients with these inflammatory myopathies, changes in Ro52/IgG/HLA-DR complex antibody titers correlated with changes in KL-6 and CK levels. This suggests that Ro52/IgG/HLA-DR complex antibodies may be involved in the pathogenesis of dermatomyositis.

These findings suggest that misfolded protein/MHC class II complexes play an important role in the pathogenesis of autoimmune diseases.

## Day 2

# Session II: Clinical Insights of Dermatomyositis

---

### 1. Autoantibodies and clinical phenotypes in dermatomyositis

Manabu Fujimoto

Department of Dermatology, Osaka University Graduate School of Medicine, Japan

Dermatomyositis and antisynthetase syndrome are among idiopathic inflammatory myopathies that have skin lesions. The discovery and characterization of myositis-specific and associated autoantibodies for recent decades have been a great advance in the field of myositis, as they are closely correlated with the clinical and histopathological phenotypes, internal complications such as interstitial lung disease and cancer, and treatment responses. Dermatomyositis-specific antibodies include anti-Mi-2, anti-melanoma differentiation-associated protein 5 (MDA5), anti-nuclear matrix protein 2 (NXP2), anti-transcriptional intermediary factor 1 (TIF1), and anti-small ubiquitin-like modifier activating enzyme (SAE). Patients with antisynthetase autoantibodies, especially anti-PL-7 and anti-EJ antibodies, can also present cutaneous symptoms and thus can be also diagnosed as having dermatomyositis, depending on the definition. Importantly, these autoantibodies are closely associated with distinct skin manifestations. Knowledge of various cutaneous signs and their associations with autoantibodies can help clinicians not only to diagnose DM patients but also predict their disease types even before blood testing.

# Day 2

## 2. Disease Presentations in Dermatomyositis Patients with Skin of Color

Michelle Lee, BA[1], L. Steven Brown,[2] Richard D. Sontheimer,[3] Benjamin F. Chong[1]

[1]Department of Dermatology, University of Texas Southwestern Medical Center, Dallas, TX, USA
[2]Department of Health Systems Research, Parkland Health, Dallas, TX, USA
[3]Department of Dermatology, University of Utah Health, Salt Lake City, UT, USA

Prior dermatomyositis (DM) cohort studies have not had sufficient representation of patients with skin of color (SOC). Our study investigated differences in clinical presentations and co-morbidities of DM patients without SOC (i.e. White Non-Hispanic), and with SOC (e.g. Black, Hispanic, Asian). We conducted a retrospective cohort study of 130 DM patients seen in outpatient dermatology clinics at University of Texas Southwestern and Parkland Health between January 2006 to October 2022. Compared to patients without SOC (N=68), DM patients with SOC (N=62) were younger at initial visit (median age: 44.5 vs. 54.5 years, $p<0.0001$), had longer disease duration (6.5 vs. 14.5 months, $p=0.002$), and was more predominantly female (95% vs. 82%, $p=0.02$). DM patients with SOC also were more likely to have classical DM (73% classical vs. 57% classical, $p=0.07$), poikiloderma in the upper back (42% vs. 25%, $p=0.04$), elevated muscle enzymes (77% vs. 46%, $p=0.0005$), and positive MRIs of the deltoid/femur (86% vs. 46%, $p=0.04$) versus DM patients without SOC. DM patients with SOC presented less often with linear extensor erythema of the digits (13% vs. 38%, $p= 0.001$), calcinosis cutis (16% vs. 1%, $p=0.003$), and internal malignancies (8% vs. 21%, $p=0.04$). Multivariable analysis identified younger age at initial visit (odds ratio (OR)=0.936/year, 95% confidence interval (CI) (0.904-0.969), $p<0.001$), classical DM subtype (OR=2.416 (CI: 1.048-5.572), $p=0.04$), and absence of linear extensor erythema (OR=4.293 (CI: 1.621-11.370), $p=0.003$) as significant predictors of DM patients with SOC. Our observations that DM patients with SOC had more muscle involvement and were younger suggest greater disease severity and chronicity in these patients.

# Day 2

## Session III: Treatment of Dermatomyositis

### 1. Novel DM targets

David Fiorentino, MD, PhD

Department of Dermatology, Stanford University School of Medicine, CA, USA

Dermatomyositis (DM) is a multi-systemic autoimmune disorder which typically affects the skin and muscle, and it can variably affect other organ systems, including lungs and joints. Because relatively little is known regarding pathogenesis of DM, treatment generally involves broad immunosuppression with traditional immunomodulatory agents. The presence of antigen-specific autoantibodies in DM has suggested that B cells (and, by implication T cells) are involved in DM pathogenesis and hence most of the currently employed therapies target these cells and/or their products. It is now clear that the innate immune system may play a critical role in pathogenesis, given the broad evidence for increased interferon (IFN) activation in blood and involved target tissues of DM. Novel therapies are currently (or soon to be) in the process of evaluation in clinical trials. Additionally, increasing knowledge of disease pathogenesis as well as therapeutics in other autoimmune diseases are providing other options for potential therapy. Future regulatory hurdles still exist for these strategies not only to be realized as commercially available treatments, but also to ensure that these options be made available to the entire gamut of DM patients, including those with skin-predominant disease.

# Day 2

## 2. Safety and Efficacy of Lenabasum, an oral CBR2 Agonist, in Patients with Dermatomyositis: A Phase 3 Randomized, Double Blind, Placebo-Controlled Trial

Victoria P. Werth*[1,2], Barbara White[3], Nancy Dgetluck[3], Kathleen Hally[3], Scott Constantine[3], Rohit Aggarwal[4], David Fiorentino[5], Ingrid E. Lundberg[6], Chester V Oddis[7]

[1]University of Pennsylvania, Dermatology, Philadelphia, USA, [2]Corporal Michael Crescenz VAMC, Philadelphia, USA, [3]Corbus Pharmaceuticals, Norwood, MA, USA, [4]University of Pittsburgh, Dept of Rheumatology, Pittsburgh, USA, [5]Stanford U, Dermatology, Stanford University, USA, [8]Karolinska Institute, Division of Rheumatology, Dept of Medicine, Stockholm, Sweden

Safe and effective treatments are of significant unmet need in DM. Lenabasum, a CB2 agonist that activates resolution of inflammation, improved skin disease, patient-reported outcomes, and biomarkers in a Phase 2 study of DM patients with active skin disease.
The objective was to evaluate the efficacy and safety of lenabasum in a Phase 3 double-blind study in DM. DM patients ≥ 18 years old with active skin with or without muscle involvement were enrolled in 55 sites in North America, Europe, and Asia-Pacific. Stable doses of background immunosuppressant were allowed. Subjects were randomized 2:1:2 to lenabasum 20 mg BID, lenabasum 5 mg BID, or placebo BID for 52 weeks, with visits ≤ 8 weeks apart. The study was stopped after all subjects completed Week 28. Some subjects had completed Week 52 by then. The primary efficacy endpoint was Total Improvement Score (TIS) at Week 28 and a secondary efficacy endpoint was TIS at Week 52, for lenabasum 20 mg BID vs placebo. 175 subjects (69 lenabasum 20 mg BID, 35 lenabasum 5 mg BID, 71 placebo BID) received study drug; 167 completed Week 28, and 103 completed Week 52. The most common reasons for study discontinuation were study stopped by Sponsor (34.3%), withdrawal of consent (4.5%), and adverse events (AEs, 3.9%), with similar rates among groups. Baseline demographics and disease measurements were similar among groups. The primary efficacy endpoint was not met - mean (SD) TIS score was 28.3 (19.75) vs 27.2 (19.23) at Week 28 for lenabasum 20 mg BID vs placebo, $p = 0.3311$, MMRM. Week 52 values were 40.6 (16.88) vs 34.8 (19.94), $p = 0.2290$. When analyses were restricted to subjects with muscle weakness at baseline (MMT8 < 142), TIS scores and treatment differences were greater and reached nominal statistical significance at Week 40, $p = 0.0172$. Mean (SD) improvements in CDASI activity score were numerically greater but not statistically different between lenabasum 20 mg BID group vs placebo at Week 28 [-7.1 (7.76) vs -5.8 (8.88) points, $p = 0.2775$] and Week 52 [-10.0 (9.45) vs -6.2 (12.8) points, $p = 0.0932$]. When restricting analysis of participants without muscle weakness (MMT-8 = 150), improvement in CDASI activity score was greater in the lenabasum 20 mg BID group vs placebo at Week 28, $p = 0.0461$, and Week 52, $p = 0.0059$. Related TEAEs leading to withdrawal of study product were infrequent.

## Day 2

# Sponsored Luncheon Seminar II

Amgen

---

### 1. Seeking precision medicine based on the pathophysiology of Behçet’s disease

Yohei Kirino

Department of Stem Cell and Immune Regulation, Yokohama City University Graduate School of Medicine, Japan

Behçet’s disease is a recurrent inflammatory disease that can cause oral ulcers, skin mucosal lesions, and uveitis. Through genome-wide association analysis, both acquired and innate immunity have been found to be important in the pathogenesis of the disease. However, the phenotype is heterogeneous, and treatment strategies differ from patient to patient. Yokohama City University has been conducting a disease registry since 2019 to understand the current status of patients. What is known at this time is that patients have an average disease activity index BDCAF of 2 points, which is similar at Japanese institutions other than Yokohama City University and in Korea. Furthermore, patients' own assessment of disease activity on the Face scale (range 1-7) revealed an average score of 4. This indicates that disease activity remains in many Japanese patients with Behçet’s disease. Many of these patients were given current available drugs such as colchicine and prednisolone. The clinical significance of a BDCAF score of 2 or face scale of 4 is unknown at this time, but as with rheumatoid arthritis, it is necessary to develop a treat-to-target strategy and to examine whether a treatment strategy aiming for a BDCAF score of 0 would be beneficial. Apremilast, recently indicated for oral ulcers in Behçet’s disease, has evidence to reduce BDCAF and is expected to play a role in T2T. In this talk, I would like to discuss the latest developments in Behçet’s disease research in Japan.

# Day 2

## 2. IFNα producing innate cells in systemic lupus erythematosus

Sachiko Miyake [1,2], Taigai Kuga [1,2], Goh Murayama [1,2], Ken Yamaji[2], Naoto Tamura[2], and Asako Chiba [1]

1 Department of Immunology, Juntendo University Graduate School of Medicine, Japan
2 Department of Rheumatology, Juntendo University Graduate School of Medicine, Japan

Increased interferon α (IFNα) is thought to be important in the pathogenesis of systemic lupus erythematosus (SLE). TLR7 stimulation induced IFNα production only in plasmacytoid dendritic cells (pDCs). IFNα-producing capacity of lupus pDCs was enhanced and correlated with disease activity and serum IFNα levels. In contrast, cyclic GMP-AMP synthase (cGAS)-stimulator of interferon genes (STING) pathway stimulation induced monocyte and conventional DCs as well as pDCs, and monocytes constituted the main IFNα-producing cells in patients with SLE. The frequency of IFNα-producing monocytes positively correlated with SLE disease activity. STING expression and co-localization with downstream molecules including TANK-binding kinase 1 were increased in patients with SLE. Lupus monocytes upregulated senescence related genes including CDKN2A and CDKN2B and one of these genes contributed to the enhanced production of IFNα. Inhibition of the mechanistic target of rapamycin pathway suppressed monocyte IFNα production and downregulated the enhanced expression of STING and its downstream molecules in lupus monocytes.

# Day 2

## Session IV: Rheum-Derm Grand Rounds

---

### 1. (CO1) Linear cutaneous lupus erythematosus – An important differential for blaschkoid dermatoses

Ysabel Regina H. Ortiz, Juan Paolo David S. Villena, Eileen Liesl A. Cubillan

Department of Dermatology, University of the Philippines – Philippine General Hospital

Linear cutaneous lupus erythematosus (LCLE) is a rare variant of lupus that presents with unilateral erythematous papules and plaques following the lines of Blaschko. Only a few cases of LCLE have been reported, mostly in children and young adults. We describe a case of linear cutaneous lupus erythematosus in an adult Filipino female, along with our approach to diagnosis and management.

A 30-year-old Filipino female developed multiple, pruritic, erythematous papules in a linear Blaschkoid configuration on the right medial thigh and calf followed by the right arm and back over the course of 2 months. She also had a history of Raynaud phenomenon, recurrent cold urticaria, and 2 prior spontaneous abortions. She had no other cutaneous manifestations and symptoms of multiorgan involvement. The clinical diagnosis at presentation was lichen striatus with antiphospholipid antibody syndrome (APAS). Complete blood count, kidney function tests, and urinalysis were within normal range. Lupus antibodies were negative, while the antiphospholipid antibody syndrome panel was positive. Histopathology showed lichenoid-interface dermatitis with mucin deposition, consistent with cutaneous lupus erythematosus. The patient was managed as LCLE with APAS, advised sun protection and started on mometasone furoate 0.1% cream twice daily, with noted complete resolution of the linear eruption after 3 months of treatment.

To our knowledge, this is the first reported case of linear cutaneous lupus erythematosus in the Philippines. LCLE is commonly mistaken at initial presentation for other acquired inflammatory dermatoses with Blaschkoid distribution, such as lichen striatus, linear lichen planus, and linear verrucous epidermal nevus. We propose that LCLE be regarded as a primary differential in adult patients presenting with unilateral Blaschkoid dermatoses. Topical corticosteroids may be sufficient in the treatment of LCLE.

# Day 2

## 2. (CO2) Improvement of systemic sclerosis-associated digital ulcers after UVA1 phototherapy

Meagan Mandabach Olivet, BA, Kevin Yang, MD, Lauren V Graham, MD, PhD

Department of Dermatology, The University of Alabama at Birmingham

Digital ulcers represent a significant complication of systemic sclerosis that severely impair a patient’s ability to use their hands and often may lead to infection and amputation. We present the case of a 47-year-old female with history of systemic sclerosis complicated by recalcitrant ulcerations of her fingers for 12 years. Her physical exam was notable for smooth, taut skin of the hands with multiple ulcers on the bilateral finger pads and dorsal joints. Prior rheumatology management of her systemic sclerosis included hydroxychloroquine, azathioprine, mycophenolic acid, prednisone, and IVIG. In addition, she had been started on sildenafil, pentoxifylline, topical nitroglycerin ointment, as well as an initial course of UVA1 phototherapy (10 years prior to current presentation) without any improvement in digital ulcers. Given the lack of improvement with any systemic or topical therapy, UVA1 phototherapy was re-initiated given previous cases of improvement in the literature. After 20 sessions at a dose of 20-60 J/cm$^2$, the patient reported significant improvement of digital ulcers with a marked decrease in healing time. UVA1 phototherapy has previously been shown to reduce sclerosis of the skin and represents a safe modality due to its localized effect. However, treatment options for digital ulcers remain limited and reducing the burden of disease for scleroderma patients with digital ulcers is crucial for improving patients’ quality of life. Overall, UVA1 phototherapy may represent a safe and effective therapeutic option for the management of digital ulcers in systemic sclerosis.

# Day 2

## 3. (CO3) Paraneoplastic interface dermatitis presenting as exfoliative erythroderma

Hana Ahmed, MD, Lauren V Graham, MD, PhD

Department of Dermatology, University of Alabama at Birmingham, USA

We present two cases of exfoliative erythroderma with interface dermatitis and positive antinuclear antibody (ANA), initially thought to be connective tissue disease, that was ultimately determined to be paraneoplastic. A 50-year-old male, with a background of atopic dermatitis, presented with an acutely worsening rash. Physical examination showed exfoliative erythroderma with > 95% body surface area involved. Laboratory findings were significant for ANA positive at 1:320 and positive anti-topoisomerase I (Scl-70) antibody. Skin biopsy showed interface and spongiotic dermatitis with eosinophils. Concern was initially for drug-induced dermatitis vs connective tissue disease. Patient was found to have inguinal and axillary lymphadenopathy, which upon biopsy, showed marginal zone lymphoma. The other case is a 53-year old female who presented to the rheumatology/dermatology combined clinic for a concern for exfoliative erythroderma secondary to connective tissue disease with a positive ANA 1:1280 in a speckled pattern and positive anti-SSB. Upon further chart review, she had a mass on her right breast seen on ultrasound but had been lost to follow up. On physical exam, she had exfoliative erythroderma and an indurated plaque with peau d'orange changes on her right breast. Skin biopsy of the breast showed a poorly-differentiated adenocarcinoma. These two cases highlight the importance of a good history, review of systems, physical exam, and chart review for patients that present with exfoliative erythroderma initially thought to be connective tissue disease.

## Day 2

### 4. (CO4) ACLE in a young woman after Dapsone treatment

Amanda Rödöö and Filippa Nyberg

Department of Dermatology, Karolinska University Hospital. Stockholm, Sweden

## Day 2

### 5. (CO5) Dermatomyositis with minimal myositis and MI2 autoantibodies

Britta Krynitz and Filippa Nyberg

Department of Pathology and Department of Dermatology, Karolinska University Hospital. Stockholm, Sweden

## Day 2

### 6. (CO6) Cutaneous lupus erythematosus after Nivolumab treatment

Jan Lapins and Filippa Nyberg

Department of Dermatology, Karolinska University Hospital. Stockholm, Sweden

## ～ *Poster session* ～

# RP1

## DESIGN OF A GLOBAL, DOUBLE-BLIND, PLACEBO (PBO)-CONTROLLED PHASE 2 TRIAL USING DEUCRAVACITINIB (DEUC), AN ORAL, SELECTIVE, ALLOSTERIC TYROSINE KINASE 2 (TYK2) INHIBITOR, IN PATIENTS (PTS) WITH ACTIVE DISCOID AND/OR SUBACUTE CUTANEOUS LUPUS ERYTHEMATOSUS

Victoria P. Werth,[1] Joseph F. Merola,[2] Joerg Wenzel,[3] Nikolay Delev,[4] Harini Kothari,[4] Richard Meier,[4] Shalabh Singhal,[4] Malavi Madireddi,[4] Shimon Korish[4]

[1]University of Pennsylvania and the Michael J. Crescenz VA Medical Center, Philadelphia, PA, USA; [2]Brigham and Women's Hospital, Harvard Medical School, Boston, MA, USA; [3]University Hospital of Bonn, Bonn, Germany; [4]Bristol Myers Squibb, Princeton, NJ, USA

Deucravacitinib (DEUC) is a first-in-class, oral, selective, allosteric TYK2 inhibitor approved in multiple countries for adult plaque psoriasis treatment. DEUC efficacy was shown in a phase 2 trial of pts with systemic lupus erythematosus. Pts with discoid and/or subacute cutaneous lupus erythematosus (DLE/SCLE) have increased type I interferon (IFN) expression. DEUC mediates type I IFN, interleukin (IL)-12 and IL-23 signaling and may be effective in treating DLE/SCLE. This phase 2 trial (NCT04857034) will examine DEUC efficacy/safety vs PBO in pts with active DLE/SCLE. The trial is enrolling adults with biopsy-confirmed clinical DLE/SCLE. Key eligibility criteria and study design are shown (**Figure**). Eligible pts will be randomized 1:1:1 to PBO or to 1 of 2 DEUC doses for 16 weeks. At week 16, PBO pts will be re-randomized 1:1 to DEUC dose 1 or 2 until week 52. Original DEUC pts will continue treatment until week 52. The primary endpoint is percentage change in CLASI (CLASI-A) at week 16 vs baseline. The trial will also evaluate the DEUC safety/tolerability profile, exploratory endpoints, pt-reported outcomes, and pharmacodynamic parameters. Inclusion of 75 pts (25/treatment group) is planned in 8 countries in North and South America, Europe, and Asia-Pacific.

**Figure. Design**

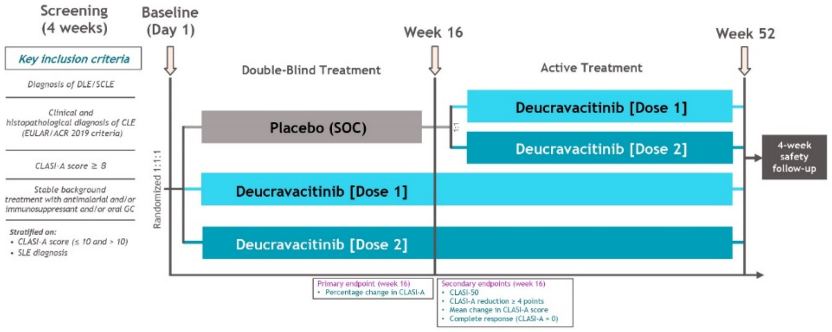


ACR, American College of Rheumatology; CLASI-A, Cutaneous Lupus Erythematosus Disease Area and Severity Index-Activity; CLASI-50 (CLASI-A-50), decrease of ≥ 50% from baseline Cutaneous Lupus Erythematosus Disease Area and Severity Index; EULAR, European Alliance of Associations for Rheumatology; GC, glucocorticoids; SOC, standard of care.

# RP2

## EFFICACY AND SAFETY OF LITIFILIMAB IN CUTANEOUS LUPUS ERYTHEMATOSUS (CLE): PHASE 2/3 AMETHYST STUDY DESIGN

Victoria P. Werth,[1] Joseph F. Merola,[2] Benjamin F. Chong,[3] Filippa Nyberg,[4] Eric F. Morand,[5] Ricardo Galimberti,[6] Wenbin Zhu,[7] Qianyun Li,[7] Jennifer Sacks,[8] Weihong Yang,[7] Michael Schindelar,[7] Catherine Barbey[9]

[1]University of Pennsylvania and Corporal Michael J. Crescenz VAMC, Philadelphia, PA, USA; [2]Brigham and Women's Hospital, Harvard Medical School, Boston, MA, USA [3]UT Southwestern Medical Center, Dallas, TX, USA; [4]Karolinska University Hospital, Stockholm, Sweden; [5]Monash University, Victoria, Australia; [6]Universidad Nacional de Buenos Aires, Department of Dermatology, Buenos Aires, Argentina; [7]Biogen, Cambridge, MA, USA; [8]Biogen, Durham, NC, USA; [9]Biogen, Baar, Switzerland

Data from the Phase (Ph) 2 LILAC study (NCT02847598) of litifilimab (BIIB059), a humanized IgG1 monoclonal antibody targeting BDCA2, supported its continued development in CLE (Werth VP, et al. N Engl J Med 2022;387:321–331).

AMETHYST (NCT05531565) is a global, multicenter, randomized, double-blind, placebo-controlled (DBPC), operationally seamless Ph 2/3 study of litifilimab. Eligible participants (pts) are ≥18 years, with a histologically confirmed diagnosis of subacute or chronic CLE (with/without systemic manifestations) refractory/intolerant to antimalarials, and with CLASI-A score ≥10. Enrolled pts will receive subcutaneous litifilimab or placebo once every four weeks (Q4W) during W0–20 and at W2; all pts will receive litifilimab Q4W during W24–48 and placebo or litifilimab (respectively) at W26 to maintain blinding. Stable lupus background treatment is permitted. Primary endpoints are the proportion of pts achieving a Cutaneous Lupus Activity–Investigator Global Assessment–Revised (CLA-IGA-R) Erythema score of 0 or 1 at W16 (Ph 2; Ph 3 in USA), or a ≥70% decrease from baseline in CLASI-A score (CLASI-70 response) at W24 (Ph 3 in rest of world). Secondary endpoints (including CLASI-50 response, change from baseline in CLASI-D score, further CLA-IGA-R analyses, and safety) will evaluate efficacy and safety during the DBPC and extended treatment periods.

## RP3

## RAPID EFFICACY OF ANIFROLUMAB IN REFRACTORY CUTANEOUS LUPUS ERYTHEMATOSUS: A PROSPECTIVE STUDY OF 11 PATIENTS WITH SYSTEMIC LUPUS

François Chasset, MD, PhD[1], Léa Jaume, MD[1], Alexis Mathian[2], MD, PhD[2], Noémie Abisror[3], Amélie Dutheil, MD[1], Annick Barbaud, MD, PhD[1], Diane Kottler[4], MD, Céline Girard MD[5], Sandrine Jousse-Joulin, MD[6], Marie Tauber, MD[7], Cristina Bulai Livideanu, MD[7], Véronique Avettand-fenoel, MD, PhD[8], Raphael Lhote, MD[2], Micheline Pha, MD[2], Zahir Amoura, MD, MSc[2]

[1]Service de Dermatologie et Allergologie, Hôpital Tenon, [2]Service de Médecine Interne 2, Groupement Hospitalier Pitié–Salpêtrière, [3]Service de Médecine Interne, Hôpital Saint-Antoine, [4]Service de dermatologie et vénérologie, CHU Caen, [5]Service de dermatologie et vénérologie, CHU Montpellier, [6]Service de rhumatologie, CHU Brest, [7]Service de dermatologie, CHU Toulouse, [8]Service de Virologie, Hôpital Cochin, France

**Background:** Clinical trials have shown anifrolumab to be effective for cutaneous lupus erythematosus (CLE) associated with systemic lupus erythematosus (SLE). However, its real-life efficacy on CLE refractory to standard therapy is unknown. **Objectives:** To assess anifrolumab efficacy in refractory cutaneous manifestations of SLE. **Methods:** multicenter prospective study enrolling SLE patients with biopsy proven active CLE refractory to at least 3 currently available CLE treatment including belimumab. The primary outcome was the proportion of partial response (PR) at week 16 defined by a decrease of CLE Disease Area and Severity Index activity of at least 50% (CLASI-A 50). Complete response (CR) defined as a CLASI-A score of 0, SLE activity (SELENA-SLEDAI score) and adverse events were also assessed. **Results:** 11 women were included. CLASI-A 50 at week 16 was reached by the 11 patients. CR was observed in 6 patients (54%). Median CLASI activity decreased from 15 (4-35) at baseline to 2 (0-13) at week 16 ($p<0.001$). Median SELENA-SLEDAI score decreased from 8 (4-22) at baseline, to 4 (0-10) at week 16 ($p=0.002$) and all patients with baseline articular involvement had a disappearance of their clinical symptoms. 8 patients had adverse events including two mild COVID-19 and one herpes zoster. **Conclusion:** Anifrolumab is a promising therapeutic option in SLE patient with refractory CLE.

# RP4

## EFFICACY AND SAFETY OF DEUCRAVACITINIB (DEUC), AN ORAL, SELECTIVE, ALLOSTERIC TYROSINE KINASE 2 (TYK2) INHIBITOR, IN PATIENTS (PTS) WITH ACTIVE SYSTEMIC LUPUS ERYTHEMATOSUS (SLE)

Victoria P. Werth,[1] Marilyn Pike,[2] Joan T. Merrill,[3] Eric Morand,[4] Ronald van Vollenhoven,[5] Coburn Hobar,[6] Nikolay Delev,[6] Vaishali Shah,[6] Brian Sharkey,[6] Thomas Wegman,[6] Ian Catlett,[6] Subhashis Banerjee,[6] Shalabh Singhal[6]

[1]Univ of Pennsylvania and Michael J. Crescenz VA Medical Center, Philadelphia, PA, USA; [2]MedPharm Consulting, Inc, Raleigh, NC, USA; [3]Oklahoma Medical Research Foundation, Oklahoma City, OK, USA; [4]Monash Univ, Victoria; Dept of Rheumatology, Monash Health, Victoria, Australia; [5]Amsterdam Univ Medical Centers, Amsterdam, the Netherlands; [6]Bristol Myers Squibb, Princeton, NJ, USA

Deucravacitinib (DEUC) is an oral, selective, allosteric TYK2 inhibitor. DEUC efficacy/safety in pts with active SLE was assessed in a 48-week, double-blind, placebo (PBO)-controlled, phase 2 trial (NCT03252587).[1] Pts on standard background medications were randomized 1:1:1:1 to DEUC (3 mg BID, 6 mg BID, 12 mg QD) or PBO. Further eligibility and study design have been described.[1] The primary endpoint was the proportion of pts achieving SLE Responder Index (SRI[4]) at week 32. Key secondary endpoints at week 48 included the proportion of pts achieving SRI(4) and ≥50% decrease from baseline (BL) Cutaneous Lupus Erythematosus Disease Area and Severity Index (CLASI-50) in pts with BL CLASI ≥10. Change from BL in CLASI Activity Score (CLASI-A) was also assessed. Of 363 pts, 76% completed 48 weeks of treatment. BL patient characteristics were balanced; BL mean CLASI-A scores ranged from 8.0–8.6. The primary endpoint was met, with significantly greater proportions of 3 and 6 mg BID DEUC pts vs PBO achieving SRI(4) responses; SRI(4) response was sustained to 48 weeks.[1] In pts with BL CLASI ≥10, significantly greater mean changes in CLASI-A were observed for DEUC vs PBO. Rates of adverse events (AEs), serious AEs, and AEs of interest were similar between DEUC and PBO groups. Deucravacitinib showed sustained, meaningful efficacy in SRI(4), improvement in mucocutaneous activity, and was well tolerated in pts with active SLE up to 48 weeks.

1. Morand E, et al. *Arthritis Rheumatol.* 2023;75(2):242-252.

# RP5

## DISEASE-RELATED EXPERIENCES OF TIKTOK™ USERS WITH LUPUS ERYTHEMATOSUS: QUALITATIVE AND CONTENT ANALYSES

Lindsey J Wanberg[1] and David R Pearson[1,2]

[1]University of Minnesota Medical School, USA

[2]University of Minnesota Department of Dermatology, USA

**Background:** Lupus erythematosus (LE) is an autoimmune disease with known detriment to quality of life. TikTok™ provides a unique opportunity to understand patient experiences with LE in a non-clinical sample. **Methods:** TikTok videos were included if they contained "#lupus", were downloadable and in English, and involved the experience of an individual with LE. A codebook was developed using an inductive approach of iterative coding until saturation was reached. Content analysis was performed within the analysis software, NVivo. Themes were derived through an inductive thematic analysis approach. **Results:** 153 TikTok videos met inclusion criteria. The most common codes were "experiences with symptoms" (69.3%) and "mucocutaneous symptoms" (39.9%), which had 24.4 and 14.1 million views, respectively. Five themes were derived: 1) Mucocutaneous symptoms had profound effects on mental health and body image of TikTok users with LE; 2) Users' negative experiences with health care workers were often derived from diagnostic delays and "medical gaslighting"; 3) Users portrayed non-pharmacologic interventions, such as diet and naturopathic remedies, positively, whereas pharmacologic treatments were referred to negatively or as "chemotherapy"; 4) LE symptoms, particularly musculoskeletal symptoms and fatigue, interfered with users' functioning; and 5) Although TikTok users had strong support systems, feelings of isolation were common and attributed to battling an "invisible illness". **Conclusion:** This study has several clinical implications. Since mucocutaneous symptoms were predominant drivers of distress, treatment of hair loss and rash is vital. However, pharmacologic therapies were depicted negatively, reinforcing the need for discussions on safety and efficacy of these treatments. Additionally, while TikTok users had robust support systems, feelings of having an "invisible illness" and "medical gaslighting" dominated negative interactions with others, underscoring the importance of providing validation for patients.

# RP6

## Usefulness of medical makeup in patients witdh cutaneous lupus erythematosus

Masatoshi Jinnin[1], Chikako Kaminaka[1,2], Fukumi Furukawa[1], Yuki Yamamoto[1,2]

[1]Department of Dermatology, Faculty of Medicine, Wakayama Medical University, Wakayama, Japan
[2]Department of Cosmetic Dermatology and Photomedicine, Faculty of Medicine, Wakayama Medical University, Wakayama, Japan

Skin diseases are more likely to cause cosmetic problems than visceral diseases, and chronic lesions in particular often damage to the quality of life (QOL) of patients for a long time. We report on the effects of medical makeup on QOL of two lupus erythematosus patients by comparing skindex-16 before and after the makeup.

# RP7

## Staphylococcus aureus skin colonization promotes SLElike autoimmune inflammation via neutrophil activation and the IL-23/IL-17 axis

Hitoshi Terui[1], Kenshi Yamasaki[1]*, Moyuka Wada-Irimada[1], Mayuko Onodera-Amagai[1], Naokazu Hatchome[1], Masato Mizuashi[1], Riu Yamashita[2], Takeshi Kawabe[3], Naoto Ishii[3], Takaaki Abe[4, 5, 6], Yoshihide Asano[1], and Setsuya Aiba[1]

[1]Department of Dermatology, Tohoku University Graduate School of Medicine; Sendai, Japan; [2]Division of Translational Informatics, Exploratory Oncology Research & Clinical Trial Center, National Cancer Center; Kashiwa, Japan; [3]Department of Microbiology and Immunology, Tohoku University Graduate School of Medicine; Sendai, Japan; [4]Division of Nephrology, Endocrinology, and Vascular Medicine, Tohoku University Graduate School of Medicine, Sendai, Japan; [5]Division of Medical Science, Tohoku University Graduate School of Biomedical Engineering, Sendai, Japan; [6]Department of Clinical Biology and Hormonal Regulation, Tohoku University Graduate School of Medicine, Sendai, Japan.

Systemic lupus erythematosus (SLE) is an autoimmune disease, but its etiology is not completely understood. The microbiota in the nasal cavity and gut are suggested to be involved in SLE development, but the influence of skin microbiota is still unclear. Here, we demonstrated that epithelial cell-specific IκBζ-deficient (*Nfkbiz*$^{\Delta K5}$) mice associated with *Staphylococcus aureus* developed SLE-associated autoantibodies and glomerulonephritis with IgG deposition. Epicutaneous *S. aureus* application significantly increased staphylococcal colonization on the skin of *Nfkbiz*$^{\Delta K5}$ mice with reduced expression of antimicrobial peptides. This promoted caspase-mediated keratinocyte apoptosis and neutrophil activation, inducing the IL-23/IL-17 immune response by activating dendritic cells and T cells. Furthermore, the administration of anti-IL-23p19 antibody and anti-IL-17A antibody alleviated the systemic autoimmune response. Our results provide a novel murine model for SLE and reveal the fundamental importance of skin microbiota controlled by epidermal IκBζ in maintaining systemic immune homeostasis.

# RP8

## Response of chronic cutaneous lupus lesions in SLE to interferon receptor blockade parallels reduction of interferon score in blood

Claudia Günther[1], Louisa Fennen[1], Christine Wolf[2], Martin Aringer[3], Min Ae Lee-Kirsch[2]

[1]Department of Dermatology, University Medical Center Hospital TU Dresden
[2]Department of Pediatrics, University Medical Center Hospital TU Dresden
[3]Division of Rheumatology, Department of Medicine III, University Medical Center Hospital TU Dresden, Germany

Cutaneous lupus has a wide spectrum of clinical manifestations, which often only partially respond to conventional therapies. Among the cytokines highly implicated in the pathogenesis of cutaneous lupus, type I interferons (IFNs) play a prominent role. Type I IFN-stimulated genes and proteins are typically upregulated in both the skin and blood of lupus patients. Type I IFN receptor blockade with anifrolumab is EMA- and FDA-approved for the treatment of SLE.

Here, we evaluated clinical efficacy of anifrolumab therapy in treatment refractory cutaneous lupus lesions in 5 SLE patients and the correlation with their type I interferon score. We treated 5 patients with cutaneous involvement of systemic lupus (2 SCLE, 1 ACLE, 2DLE) with anifrolumab. Previously refractory skin lesions showed rapid improvement with 50% reduction in median CLASI-A after one month and completely resolved within 3-6 months of treatment. Relief of cutaneous symptoms was associated with improvement of life quality. In parallel to clinical response, we observed a reduction in IFN-scores determined by measuring the mRNA expression of seven IFN-stimulated genes in blood. IFN scores kept declining over 6 to 12 months of treatment, and two patients reached levels comparably to healthy controls after 6 months. Our limited data suggest that interferon score declines slower compared to the rapid relief of skin lesions. This might indicate that the cutaneous response is not entirely dependent on systemic reduction of interferon stimulated genes in blood. In conclusion these data indicate rapid efficacy of anifrolumab in cutaneous lupus in parallel with a decline in interferon score in blood.

# RP9

## Anifrolumab for systemic lupus erythematosus: A clinical study of Japanese patients in Kanazawa University Hospital

Natsumi Fushida[1], Motoki Horii[1], Kyosuke Oishi[1], Takashi Matsushita[1]

[1] Department of Dermatology, Faculty of Medicine, Institute of Medical, Pharmaceutical and Health Sciences, Kanazawa University, Kanazawa, Japan

We investigated the effectiveness of anifrolumab treatment for systemic lupus erythematosus (SLE). We treated five patients (aged 30 to 65 years, median: 47 years, one male and four females) with SLE with anifrolumab between January to December 2022 at Kanazawa University Hospital. The period between the onset and initiation of treatment with anifrolumab was 60 to 276 months (median 228 months), and the patients' systemic lupus erythematosus disease activity index-2000 (SLEDAI-2K) before treatment was 2 to 4 (median 2). Three of the patients had skin rash or alopecia, and their cutaneous lupus erythematosus disease area and severity index (CLASI) activity score was 2 to 9 (median 4). Four of the patients could continue treatment with anifrolumab, while one could not because of uncontrolled pleurisy and pericarditis. Our results showed that anifrolumab was effective in treating SLE and reduced both SLEDAI-2K and CLASI activity score (median 100% decrease). Furthermore, we could reduce the oral corticosteroid dosage in all patients who could continue the treatment. Our investigations indicates that anifrolumab is effective not only for reducing disease and eruption activity, but also corticosteroid tapering.

# RP10

## Association between periungual changes and myositis-specific autoantibodies in patients with idiopathic inflammatory myopathies

Ikuko Ueda-Hayakawa, Emi Kaneda, Aya Maekawa, Kyoko Tonomura, Noriko Arase, Manabu Fujimoto

Department of Dermatology, Osaka University Graduate School of Medicine, Japan

Myositis-specific autoantibodies (MSAs) are detected in ~60–70% of patients with myositis. These MSAs are strongly associated with a distinct disease phenotype, allowing for MSA-based classification of myositis subsets. This study aimed to determine the association between clinical features of periungual changes in adult Japanese idiopathic inflammatory myopathies (IIM) patients and the presence of MSAs. We retrospectively examined a cohort of 78 patients with IIM. Periungual changes visible to the naked eye were assessed. An enzyme-linked immunosorbent assay testing to determine the presence of anti-melanoma differentiation-associated gene 5 (anti-MDA5), anti-aminoacyl tRNA synthetase (anti-ARS), anti-transcriptional intermediary factor 1-γ (anti-TIF1γ), or anti-Mi-2 antibodies were measured in the clinical laboratory. Erythema of lateral nailfold was most frequently observed in patients with anti-MDA5. Hemorrhage of proximal nailfold was more commonly observed in anti-ARS patients or anti-Mi-2 compared to anti-MDA5 or anti-TIF1γ groups. Blood crust or crust of proximal nailfold was most frequently observed in patients with anti-MDA5. Remarkably, blood crust or crust of lateral nailfold was observed in almost all of the patients with anti-MDA5. The patients with skin atrophy of proximal or lateral nailfold frequently belonged to the anti-TIF1γ group (8/9; 89%). Both erythema and blood crust or crust of lateral nailfold were observed in about 90% of patients with anti-MDA5, which are considerably specific to anti-MDA5 positivity. The differences in periungual changes may reflect the distinct pathogenesis among DM patients.

# RP11

## Association between the sites of skin lesions and autoantibodies in juvenile dermatomyositis

Yasushi Ototake, Tomoya Watanabe, Asami Akita, Mao Suzuki, Miwa Kanaoka, Yukie Yamaguchi

Department of Environmental Immuno-Dermatology, Yokohama City University, Japan

**Objectives:** Dermatomyositis (DM)-specific autoantibody is often detected in juvenile DM (JDM), however, studies showing the association of these antibodies and skin lesions in JDM are limited. In this study, we examined characteristics of the sites of cutaneous involvement according to autoantibodies. **Methods:** Thirty-six patients with JDM were retrospectively analyzed at Yokohama City University Hospital from 2010 to 2021. Age, sex, DM-specific autoantibodies (anti-MDA5, TIF1γ, Mi2, ARS antibodies), organ involvements (interstitial lung disease (ILD) and muscle), and skin manifestation were collected from medical records and photographs. **Results:** The cohort consisted of 14 males and 22 females, and mean age at onset of JDM was 7.1 ± 4.1 years. DM-specific autoantibody was identified in 24 patients (anti-MDA5: 12, anti-TIF1γ: 10, anti-Mi2: 2, anti-ARS: 0). The age of onset was significantly older in anti-MDA5 antibody positive patients (9.6 ± 4.3 years, $P < 0.05$). ILD was observed in 8 patients (22%) and all patients were positive for anti-MDA5 antibody. Muscle involvement was observed in 30 patients (83%). Inverse Gottron's sign ($P < 0.05$) was significantly seen in anti-MDA5 positive patients, and poikiloderma was seen in anti-TIF1γ positive patients ($P < 0.05$). Furthermore, we divided the facial erythema into 6 sites and analyzed the differences between autoantibodies. Interestingly, erythema on cheekbone site ($P < 0.05$) was significantly observed in anti-MDA5 antibody positive patients while eyebrow ($P < 0.001$) and forehead ($P < 0.001$) were significantly observed in anti-TIF1γ antibody positive patients. Furthermore, patients with erythema on cheekbone ($P < 0.01$) and inner canthus site ($P < 0.05$) were significantly associated with ILD. **Conclusion:** These results suggested that DM-specific autoantibody can be predicted from specific skin sites. Furthermore, erythema on inner canthus and cheekbone site may be danger signal for ILD. The assessment of skin sites may lead to earlier diagnosis and treatment for JDM.

# RP12

## Evaluation of apremilast, an oral phosphodiesterase 4 inhibitor, for refractory cutaneous dermatomyositis: A Phase 1b clinical trial

Risa Konishi[1,2], Ryota Tanaka[2], Sae Inoue[2], Yuki Ichimura[1], Toshifumi Nomura[2], Naoko Okiyama[1]

[1]Department of Dermatology, Graduate School of Medical and Dental Sciences, Tokyo Medical and Dental University, Japan
[2]Department of Dermatology, Faculty of Medicine, University of Tsukuba, Japan

Dermatomyositis, an idiopathic inflammatory myopathy, is characterized by cutaneous itchy manifestations that impair the quality of life of patients and are frequently refractory and recurrent even after intensive immunosuppressive treatments. To evaluate the effectiveness and safety of apremilast, an oral phosphodiesterase-4 inhibitor, in treating refractory skin-dominant dermatomyositis, we performed this prospective, single-arm, interventional study. A total of 5 Japanese patients (1 male and 4 females; median [range] age, 64 [37-71] years) with refractory dermatomyositis-associated cutaneous manifestations were recruited and treated with a 12-week course of oral apremilast. Their myositis and interstitial lung disease were absent or in remission. Among 5 enrolled patients, 4 patients experienced diarrhea and/or vomiting, 2 of which withdrew from the study and recovered quickly afterwards, and the residuary 2 of which recovered during continued treatment. A total of 3 evaluable female patients received apremilast treatment for 12 weeks. A 39.4% reduction from baseline cutaneous dermatomyositis disease area and severity index (CDASI) total activity score, but not the damage score, at week 12 was observed in all 3 patients. Visual analogue scale (VAS) of itching, and quality of life by dermatology life quality index (DLQI) were improved in 1 and 2 apremilast-treated patients, respectively. Apremilast can be suggested as a possible treatment for skin-dominant dermatomyositis when intensive immunosuppressive therapies for myositis and interstitial lung disease are not required.

# RP13

## Leukemia inhibitory factor is a potential marker for anti-melanoma differentiation-associated gene 5 antibody-positive dermatomyositis with interstitial lung disease

Yuki Ichimura[1,2], Risa Konishi[1,2], Toshifumi Nomura[2], Naoko Okiyama[1]

[1]Department of Dermatology, Graduate School of Medical and Dental Sciences, Tokyo Medical and Dental University, Japan
[2]Department of Dermatology, Faculty of Medicine, University of Tsukuba, Japan

Anti-melanoma differentiation-associated gene 5 (MDA5) antibody-positive dermatomyositis (DM) frequently show rapidly progressive interstitial lung disease (RP-ILD), etiology of which remains unclear. RNA sequencing analyses of an autopsy lung sample from a 74-year-old woman with anti-MDA5 antibody-positive RP-ILD was performed in comparison to an age- and sex-matched normal lung sample. From this RNA dataset, gene ontology enrichment analysis in 519 differential expressed genes showed a strong association with antigen binding. Protein-protein interaction analysis identified several upregulated modules including the gene sets of lung surfactant molecules, major histocompatibility complex II-related molecules, collagen formation, and Interleukin (IL)-4 and IL-13 signaling. Moreover, Remarkable upregulated expressions of several inflammatory cytokines including IL-1β, IL-6, and leukemia inhibitory factor (LIF) were also detected. Next, we measured the levels of LIF in the serum samples from 12 cases of anti-MDA5 antibody-positive ILD, 12 cases of anti-aminoacyl tRNA synthetase (ARS) antibody-positive ILD, 10 cases of anti-transcription intermediary factor 1γ/anti-Mi-2 antibody positive DM, and 12 healthy volunteers. Serum LIF levels were significantly elevated in anti-MDA5 antibody-positive ILD cases (median [interquartile range], 32.4 [13.2-125.7] pg/ml) when compared with anti-ARS antibody-positive ILD cases (4.9 [3.1-19.7] pg/ml, $P < 0.05$) and other DM cases (5.3 [3.9-9.7] pg/ml, $P < 0.05$). These results suggested that upregulation of LIF might be a new potential disease marker specific for anti-MDA5 antibody-positive DM with ILD.

# RP14

## Long-term nailfold video capillaroscopy findings vary by myositis-specific autoantibody in idiopathic inflammatory myopathy

Yasuhito Hamaguchi[1], Naoki Mugii[2], Motoki Horii[1], Natsumi Fushida[1], Tomoyuki Ikeda[1], Kyosuke Oishi[1], Takashi Matsushita[1]

[1]Department of Dermatology, Kanazawa University; [2]Department of Rehabilitation, Kanazawa University Hospital, Japan

Nailfold video capillaroscopy (NVC) abnormalities are the hallmark in idiopathic inflammatory myopathy (IIM). The object of this study is to assess the long-term changes in NVC findings in patients with myositis-specific antibodies (Abs) including anti-MDA5, anti-TIF1, and anti-ARS Abs. This study included 71 patients with IIM (25 patients with anti-MDA5 Abs, 24 with anti-TIF1 Abs, and 22 with anti-ARS Abs). NVC findings included giant, enlarged, and reduced capillaries, hemorrhages, capillary ramification, disorganization of the vascular array, and capillary loss. NVC findings were compared from baseline to after disease activity stabilization. The frequency of enlarged capillaries was significantly higher in patients with anti-TIF1 Abs compared to those with anti-ARS Abs (88% vs 55%, $p<0.05$). Reduced capillaries were significantly increased in patients with anti-TIF1 Abs compared to those with anti-MDA5 (96% vs 44%, $p<0.0001$) or anti-ARS Abs (96% vs 50%, $p<0.0005$). Both enlarged and reduced capillaries improved after stabilization in patients with anti-MDA5 Abs ($p<0.0001$ and $p<0.05$, respectively). These improvements were not observed in patients with anti-TIF1 and anti-ARS Abs. However, a significant reduction in hemorrhages was observed in all three groups ($p<0.0001$ for each group). In patients with anti-MDA5 Abs, enlarged and reduced capillaries and hemorrhages were associated with an improvement in clinical parameters. Conversely, in patients with anti-TIF1 and anti-ARS Abs, hemorrhages were associated with an improvement in clinical parameters, but enlarged and reduced capillaries were not. These results demonstrate that long-term changes in NVC findings may vary by myositis-specific Ab subtype. Therefore, it is important to assess individual NVC findings separately, since each finding may impact disease activity in a different manner.

# RP15

## Obesity is an Independent Risk Factor for Cancer Development Following Diagnosis of Dermatomyositis

Astia Allenzara[1], Nakisa Sadeghi[2], Carolina Alvarez[1], Steve Maczuga[3], Matthew Helm[3], Nancy Olsen[4], Amanda Nelson[1], Galen Foulke[3,5]

[1]University of North Carolina, Division of Rheumatology, Allergy and Immunology, Chapel Hill, NC; [2]University of North Carolina, School of Medicine, Chapel Hill, NC [3]Penn State Health Milton S Hershey Medical Center, Department of Dermatology, Hershey, PA; [4]Penn State Health Milton S Hershey Medical Center, Division of Rheumatology, Hershey, PA; [5]Penn State Health Milton S Hershey Medical Center, Department of Public Health Science, Hershey, PA

Patients with dermatomyositis (DM) are at an increased risk of cancer development, especially around the time of diagnosis of DM. Obesity is also a risk factor in the general population for cancer development. This study aimed to assess the association between cancer in patients with DM with and without obesity as defined by ICD code and BMI data utilizing the TriNetX platform, a deidentified dataset containing claims data for >250 million Americans. In this cross-sectional analysis of patients with DM, logistic regression modeling of the odds of cancer outcome was performed for patients with DM and obesity compared to those without obesity, adjusted for covariables. A total of 12,722 patients with DM were identified, of whom 6,055 had available BMI data. DM patients who were obese at any point had significantly higher odds 1.98 (95% Confidence interval 1.70, 2.30) of a subsequent cancer diagnosis. This association was also found in the analysis by BMI where patients with obesity (BMI greater than 30 kg/m$^2$) had an increased odds of cancer 1.23 (1.02, 1.49) when compared to normal weight patients with DM. Overall, the most frequent type of cancer was breast cancer, however patients with DM and obesity had higher frequencies of lymphoma, colorectal, prostate, melanoma, uterine, renal cancers compared to patients with DM without obesity.

# RP16

## Clinicopathological analysis 41 cases of mechanic’s hand associated with dermatomyositis

Misaki Kusano, Tatsuhiko Mori, and Toshiyuki Yamamoto

Department of Dermatology, Fukushima Medical University, Japan

Dermatomyositis is one of the autoimmune diseases that presents with various cutaneous symptoms. Mechanic’s hand and Gottron's sign are representative cutaneous findings in dermatomyositis, but their histopathological differences have not yet been clarified. We analyzed the clinical symptoms of dermatomyositis patient mechanic’s hand who had visited our department between 2010 and 2022. A histological examination was carried out in 41 cases, which revealed hyperkeratosis in all cases and liquefaction degeneration in the epidermis in 31 cases. The male to female ratio was 9:32 and the average age was 54.1 years. Also, we compared skin biopsy specimens from 15 dermatomyositis patients with mechanic’s hand and Gottron’s sign who visited our department. Hematoxylin and eosin staining revealed hyperkeratosis in 14 of 15 biopsied specimens of Gottron's sign, and in all biopsied specimens of mechanic’s hand. We discuss clinical findings and pathological histology of mechanic’s hand special staining for MxA, CD123, S100A8 and S100A9.

# RP17

## A study on regaining gait independence in patients having idiopathic inflammatory myopathy

Naoki Mugii[1], Pleiades Tiharu Inaoka[2], Yasuhito Hamaguchi[3], Takashi Matsushita[3]

[1]Department of Rehabilitation, Kanazawa University Hospital, Japan
[2]School of Health Science, Kanazawa University, Japan
[3]Department of Dermatology, Faculty of Medicine, Institute of Medical, Pharmaceutical and Health Sciences, Kanazawa University, Japan

Patients with idiopathic inflammatory myopathy (IIM) may present severe gait disorder, but there are no detailed reports on regaining gait. This study aims to clarify the functional prognosis of IIM patients with severe gait disturbance. From 2004 to December 2022, among 231 adult IIM patients referred to our hospital's rehabilitation department, 23 patients (17 females and six males) whose muscle strength could be evaluated before the start of treatment and who could not walk independently during the course of treatment were included in this study. Subjects had an average age of 59.3 years and an average disease duration of 2.6 months. Manual muscle test at initial evaluation, myositis-specific antibodies, Barthel Index (BI), the period until reacquisition of independent gait after treatment was retrospectively investigated. Approval (No.960) was obtained from the university's ethics committee for the research. The median MMT muscle strength before treatment was 2 in the anterior neck flexor, 2 in the deltoid, 2 in the iliopsoas, and 4 in the quadriceps femoris. Myositis-specific antibodies were positive in 18 cases, including anti-aminoacyl tRNA synthetase antibody in 3 cases, anti-transcription intermediary factor 1 in 5 cases, anti-signal recognition particle antibody in 3 cases, anti-melanoma differentiation-associated gene 5 antibody in 2 cases, anti-Mi-2 antibody in 2 cases, anti-nuclear matrix protein -2 antibody in 2 cases. One case was positive for anti-3-hydroxy-3-methylglutaryl-coenzyme A reductase antibody. The average BI was 45.4 points at the initial assessment, 33.0 points at the lowest, and 94.0 points at the final evaluation, and the average time to regain walking was 2.9 months (0.5-6 months). Even if severe gait disturbance occurred in IIM, it was possible to regain gait within six months regardless of the type of myositis-specific antibody.

# RP18

## Effect of previous rituximab administration on immune response after SARS-CoV-2 vaccination in Japanese patients with systemic sclerosis (SSc)

Ruriko Kawanabe, Ayumi Yoshizaki, Kazuki M. Matsuda, Hirohito Kotani, Teruyoshi Hisamoto, Yuta Norimatsu, Ai Kuzumi, Takemichi Fukasawa, Satoshi Ebata, Asako Yoshizaki-Ogawa, and Shinichi Sato

Department of Dermatology, University of Tokyo, Japan

Previous studies have shown that anti-CD20 antibody therapy blunts the immune response to SARS-CoV-2 vaccine. To determine the effect of anti-CD20 antibody, rituximab on the humoral immune response to SARS-CoV-2 vaccine in Japanese patients with systemic sclerosis (SSc), we collected serum samples of 30 SSc patients (15 with a history of rituximab administrations, 15 without a history of rituximab administrations) 2 weeks to 3 months after the second dose of SARS-CoV-2 mRNA-based vaccine and quantified for anti-SARS-CoV-2 spike (S) IgG. As a result, anti-S IgG was detected in 5 of 15 patients (33.3%) who had received rituximab while in 12 of 15 patients (80.0%) who had not received rituximab ($p<0.05$). Anti-S IgG levels were significantly lower in patients who had received rituximab (median 0 AU/ml; range 0-153 AU/ml) than in patients who had not received rituximab (median 25.6 AU/ml; range 0-202 AU/ml) ($p<0.01$). In multivariate models, comparing accumulated rituximab dose (g), mycophenolate mofetil (MMF) (mg/day), prednisolone (mg/day), and the time between vaccination and testing (days), accumulated rituximab dose and MMF had a significant effect on anti-S IgG positivity (odds ratio [OR]: 0.635, 95% confidence interval [CI]: 0.430-0.937, $p<0.05$, OR: 0.997, 95% CI: 0.995-0.999, $p <0.05$, respectively). It was shown that rituximab administration significantly attenuates humoral immune response after SARS-CoV-2 vaccination in Japanese SSc patients. Furthermore, MMF can influence the immune response after SARS-CoV-2 vaccination as well.

# RP19

## BONE DENSITY AND FRACTURE RISK IN PEDIATRIC AND ADULT PATIENTS WITH LINEAR MORPHEA OF THE EXTREMITY: A RETROSPECTIVE REVIEW

Maha Kazmi[1], Bianca Obiakor[2], Winnie Fan[2], Rebecca Jacobson[1], Jocelyn Gandelman[1], Kelly M. Cordoro[1], Anna Haemel[1]

[1]Department of Dermatology, University of California, San Francisco, San Francisco, CA
[2] University of California, San Francisco School of Medicine, San Francisco, CA

Linear morphea of the extremities is associated with musculoskeletal complications such as limb length discrepancy and atrophy. We hypothesize that changes in structure and mobility of morphea-affected limbs (MAL) in conjunction with systemic corticosteroid exposure pose an increased risk of limb fracture over time. We retrospectively reviewed records of 21 patients from Pediatric Dermatology and 27 from Adult Dermatology at UCSF between 2015-2022; patients transitioning to adult care were analyzed as adults. Thirteen (61.9%) of 21 pediatric patients received systemic steroids in contrast to 11 (40.7%) of 27 adults. Four (14.8%) of 27 adults had limb fracture, with fractures in 3 (75%) of these 4 cases involving MAL. Comparatively, 2 (9.5%) of 21 pediatric patients had traumatic limb fractures in non-MAL. Three (11.1%) of 27 adults had DEXA scans, with 2 (66.7%) of 3 scans notable for osteopenia in MAL only, compared to non-MAL. Although the pediatric group had no DEXA scans available, 2 (9.5%) of 21 patients had osteopenia involving MAL, as seen on x-ray and MRI. The average latency period between morphea onset and MAL fracture in adults was 12 years compared to 12 and 3.5 years for non-MAL fracture in adults and pediatrics, respectively. While there may be a trend toward fracture of MAL over non-MAL in adults and often with minimal trauma, similar trends were not observed in children where traumatic fractures of non-MAL were more common. This sample size is small, and further investigation of local bone health and fracture risk in linear morphea across the lifespan is warranted to clarify risk of long-term orthopedic complications. Otherwise, a long latency between disease onset and the accumulation of structural changes increasing fracture risk may limit the opportunity for early intervention, anticipatory guidance, and monitoring.

# RP20

## Evaluation of skin hardness of patients with systemic scleroderma using SOFTGRAM

Hiraku Kokubu[1], Yasuaki Ikuno[1], Kazuyuki Uchiyama[1], Miwa Kato[1], Mayuka Yamamoto[1], Haruki Asada[1], Satona Rikitake[1], Yoshimichi Kobayashi[1], Yudai Tsukamoto[1], Takahiro Koike[1], Syuji Sugiura[1], Yasuhiro Maeda[1], Takuma Hayami[1], Kensuke Yoneta[1], Toshifumi Takahashi[1], Bunpei Yamamoto[1], Takeshi Kato[1], Yoshito Kunisaki[2], Makoto Nakatani[2], Kohei Okamoto[2] and Noriki Fujimoto[1]

[1] Department of Dermatology, Shiga University of Medical Science, Japan
[2] ISHIDA MEDICAL CO., LTD., Japan

Modified Rodnan's total skin thickness score (mRSS) has been widely used for scoring the skin hardness of patients with systemic sclerosis (SSc) for a long time since 1990s. However, it is a semiquantitative scoring system ranging from 0 (normal) to 3 (severe). Other quantitative and objective methods for the measurement of skin hardness is desired. So, we aimed this study to verify whether we could apply a measuring equipment called SOFTGRAM (SHINKO DENSHI CO., LTD., Tokyo, Japan) for the evaluation of skin hardness, which is an elastic modulus of the skin using a principle of tuning fork. Japanese technology leads the world in the field of tactile sensor, and SOFTGRAM received an award by Going-Global Innovations Competition 2019. In this study, we analyze the association between the data measured by SOFTGRAM and mRSS for 20 patients with SSc and 20 healthy controls.

# RP21

## Epitope spreading of anti-RNA polymerase III antibody response in systemic sclerosis

Hirohito Kotani[1], Kazuki M. Matsuda[1], Kei Yamaguchi[2,3], Koji Ogawa[2,3], Asako Yoshizaki-Ogawa[1], Naoki Goshima[2,3], Shinichi Sato[1], and Ayumi Yoshizaki[1]

[1]Department of Dermatology, University of Tokyo, Japan
[2]Molecular Profiling Research Center for Drug Discovery, National Institute of Advanced Industrial Science and Technology.
[3]ProteoBridge Corporation.

Systemic sclerosis (SSc) is an intractable disease characterized by fibrosis, vasculopathy, and autoimmune abnormalities as its three main features. Although the etiology and pathogenesis of SSc remain poorly understood, disruption of tolerance to autoantigens is considered important in the pathogenesis of SSc because the type of detected autoantibodies reflects the symptoms and prognosis of the disease.

When B cells encounter a foreign antigen, they produce antibodies that efficiently eliminate the antigen via epitope spreading during cognitive interaction with T cells. In autoimmune diseases, the involvement of epitope spreading in their pathogenesis is also assumed, since autoantibody levels may correlate with clinical symptoms. Nevertheless, epitope spreading to autoantigens in SSc patients has not been fully studied in the past.

In this study, we used RNA polymerase III (RNApIII) antigen, one of the autoantigens of SSc, to investigate the epitopes recognized by autoantibodies in SSc patients. The RNApIII antigen, commonly used for diagnosis in daily clinical practice, is only one part of the subunit RPC1. In our study, we synthesized all subunits of the RNApIII complex and used them to examine the reactivity of anti-RNApIII antibodies in each patient.

The results revealed that the antigen recognition sites of autoantibodies present in sera varied among patients. Furthermore, the increase in the number of RNApIII antigen subunits recognized by autoantibodies correlated with disease severity. These findings demonstrate the involvement of epitope spreading of autoantibody response in the progression of SSc using patient specimens, suggesting that the measurement of epitope spreading in SSc is a novel marker of disease severity.

# RP22

## Clinical features of patients with systemic sclerosis positive for anti-SS-A antibody: A cohort study of 156 patients

Tomoya Watanabe[1)], Yasushi Ototake[1)], Asami Akita[1)], Mao Suzuki[1)], Miwa Kanaoka[1)], Jun Tamura[2)], Yusuke Saigusa[2)], and Yukie Yamaguchi[1)]

1) Department of Environmental Immuno-Dermatology, Yokohama City University
2) Department of Biostatistics, Yokohama City University, Japan

Object**:** Anti-SS-A antibody (SSA), which is a diagnostic marker of Sjögren's syndrome (SS), is often detected in patients with systemic sclerosis (SSc). Some SSA-positive SSc patients are complicated with SS, while some do not. In this study, we retrospectively investigated the clinical characteristics of SSc patients with SSA. Methods: Retrospective chart reviews were performed of 156 patients with SSc at Yokohama City University Hospital from 2018 to 2021. Results: The cohort consisted of 18 males and 138 females. Thirty-nine patients were classified as dcSSc and 117 patients as lcSSc. Anti-centromere antibody was positive for 72 patients; anti-topoisomerase I antibody for 27; anti-RNA polymerases III antibody and anti-U1RNP antibody for 14, respectively. Forty-four patients were positive for SSA. Among them, 24 patients fulfilled the criteria for SS. The proportion of patients with interstitial lung disease (ILD), digital ulcer (DU)), and gastroesophageal reflux disease (GERD) in SSA positive group were higher than those in SSA-negative group. Multivariate analysis using LASSO revealed that SSA was statistically associated with ILD (OR = 2.67; 95% CI, 1,14-6.3; $P = 0.024$). Meanwhile, the risk for the development of GERD (OR = 1.73; 95% CI, 0.79-3.81; $P = 0.170$) and DU (OR = 2.18; 95% CI, 0.99-4.82, $P = 0.054$) was also increased by SSA although the differences were not statistically significant. Furthermore, in the analysis for 44 SSc patients with SSA, multivariate regression analysis indicated that SSA-positive SSc patients without SS is strongly associated with the proportion of dcSSc compared to those with SS (OR = 6.45; 95% CI, 1.23-32.60; $P = 0.024$). Conclusions: The presence of SSA in SSc patients increases the risk of organ involvement such as ILD and DU. Among them, the population of SSA-positive SSc without SS may have a more severe skin sclerosis.

# RP23

## A calpain inhibitor ALLN alleviates bleomycin-induced skin and lung fibrosis

Hiroshi Kasamatsu[1], Takenao Chino[1], Takumi Hasegawa[1], Natsuko Utsunomiya[1], Akira Utsunomiya[1], Masami Yamada[2], Noritaka Oyama[1], and Minoru Hasegawa[1]

[1]Department of Dermatology, University of Fukui; [2]Department of Cell Biology and Biochemistry, University of Fukui, Japan

Systemic sclerosis (SSc) is a connective tissue disease representing fibrosis in the skin and internal organs such as lungs. An activated differentiation of local progenitor cells to myofibroblasts is likely a key mechanism underlying overproduction of extracellular matrix and resultant tissue fibrosis in SSc. Calpains are family members of $Ca^{2+}$-dependent cysteine proteases for which the biological action may contribute to fibrosis in various organs. However, the precise mechanism of calpain-dependent fibrosis and therapeutic utility of their inhibitors in SSc remain unclear. This study aimed to investigate if a potent calpain inhibitor ALLN could exert antifibrotic effects on cultured human dermal and lung fibroblasts in a bleomycin-induced SSc mouse model. Normal human dermal and lung fibroblasts pretreated with ALLN were stimulated with recombinant TGF-β1, followed by assessment for expression properties of TGF-β1/Smad signaling and fibrogenic molecules. ALLN (3mg/kg/day) was intraperitoneally administered 3 times a week in bleomycin-induced SSc model mice. ALLN treatment significantly inhibited over-phosphorylation and nuclear transport of Smad2/3 in TGF-β1-stimulated dermal fibroblasts. TGF-β1-dependent increase of α-smooth muscle actin, collagen type 1, fibronectin 1, and representative mesenchymal markers were attenuated in mRNA and protein expression by ALLN. Likewise, ALLN reverted a TGF-β1-dependent change of epithelial/mesenchymal markers in human lung epithelial cells. Consistent with these, ALLN remarkably suppressed the development of skin and lung fibrosis, following decrease of infiltrating $CD3^{+}$T cells, in bleomycin-induced SSc model mice. No obvious side effects were observed. Our data provide evidence that calpains may be a primary contributor and novel therapeutic target for skin and lung fibrosis in SSc, with a treatment perspective of its inhibitor ALLN.

# RP24

## Predictive Factors for the Clinical Course of Early-Onset Severe Cases with Systemic Sclerosis in Japan: A Multicenter Prospective Observational Study

Saori Uesugi-Uchida1, Minoru Hasegawa1, Takashi, Matsushita2, Takahiro Tokunaga3 Manabu Fujimoto4 and Ministry of Health, Labour and Welfare's Systemic Sclerosis Research Group

[1]Department of Dermatology, University of Fukui, Japan; [2]Department of Dermatology, Kanazawa University, Japan; [3]Medical Research Support Center, University of Fukui Hospital, Japan; [4]Department of Dermatology, Osaka University, Japan

We aimed to assess the clinical and laboratory parameters of Japanese early systemic sclerosis (SSc) cases with diffuse cutaneous involvement (dcSSc) and/or interstitial lung disease (ILD) to identify factors that predict the later severity of clinical features. A total of 115 cases were enrolled in 10 medical centers for the first four consecutive years of follow-up. The cohort comprised 87 females and 28 males, aged 39.5-59.0 (median, 52 years). The median disease duration at registration was 22 months. Disease phenotype included 90 dcSSc with or without ILD and 25 limited cutaneous SSc with ILD, of whom 73 and 14 were positive for anti-topoisomerase I (topo I) antibody and anticentromere antibody, respectively. Treatment variations included vasodilators in 76 and systemic steroids in 81, and intravenous cyclophosphamide pulse therapy in 26 cases over four years. The initial median modified Rodnan total skin thickness score (mRSS) was 19, which finally improved to 9. The mRSS at Year 4 correlated well with baseline mRSS and fist closure. Percent vital capacity (%VC) at Year 4 (median, 91.0%) met positive and negative associations with initial %VC (median, 96.4%) and anti-topo I antibody, respectively. The frequency of digital ulcer (DU) development within four years (33.9%) was associated with the initial existence of DU (17.4%), impaired fist-closure, pitting scar, and the existence of anti-topo I antibody. HAQ-DI at Year 4 (median, 0.125) showed a positive and negative association with the initial HAQ-DI (median, 0.125) and %VC, respectively. Our data highlight the initial clinical factors that predict the subsequent progress in early-onset SSc. Notably, the value of fist closure, which reflects impaired flexion of the fingers, correlates with subsequent mRSS and the frequency of DU.

# RP25

## Expression of collagen-related piRNAs in cultured dermal fibroblasts derived from patients with systemic sclerosis

Minako Tanaka[1], Yutaka Inaba[1], Azusa Yariyama[1], Yumi Nakatani[1], Kayo Kunimoto[1], Chikako Kaminaka[1], Yuki Yamamoto[1], Masatoshi Jinnin[1]

[1]Department of Dermatology, Wakayama Medical University, Wakayama, Japan

PIWI-interacting RNAs (piRNAs) is one of the recently discovered small non-coding RNAs. piRNAs interact with PIWI proteins, bind to specific targets, and finally recruit chromatin modifiers to enable transcriptional repression. Abnormal expression of PIWI proteins and piRNAs has been described in some human cancers, with participation of some PIWI/piRNAs complexes in tumorigenesis and association with cancer prognosis. Their expression in patients with fibrotic diseases including systemic sclerosis (SSc) has not been widely elucidated. As a result, no difference was found in the PIWIL1-4 levels between normal and cultured SSc dermal fibroblasts. Among piRNAs predicted to target SSc-assciated molecules, we first found significant piR-32368 up-regulation in SSc dermal fibroblasts. Forced piR-32368 overexpression in normal fibroblasts significantly reduced COL1A1 expression both at mRNA and protein levels, but did not affect COL1A2. Accordingly, piR-32368 overexpression in SSc fibroblasts may be the negative feedback against collagen up-regulation, which could suggest the potential of piRNAs as a therapeutic target.

# RP26

## Characteristics of the cutaneous manifestations of Sjögren's syndrome in 68 Japanese patients

Satoru Arai[*], Minori Otake[*], Mitsuki Takamizawa[*], Yukari Zenke[*], Sumiyuki Mii[*]

*Department of Dermatology, St. Luke’s International Hospital, Tokyo, Japan.

Sjögren's syndrome is one of the most common autoimmune diseases. Although dry eyes and xerostomia are its main complaints, some patients may present to the dermatology department with cutaneous symptoms. Herein, we investigated the characteristics of cutaneous symptoms in 68 patients (85 cutaneous symptoms) with Sjögren's syndrome, who visited our department between January 1, 2005 and December 31, 2022, with cutaneous symptoms as their main complaint.

The female to male ratio was 12.6:1 and the mean age of the patients was 51.0 ± 16.0 years. The most common skin condition was chilblains (17 cases), while annular erythema (10 cases), urticaria (7 cases), subacute cutaneous lupus erythematosus erythematosus (5 cases), hyper-gammaglobulinemic purpura (5 cases), blepharitis (4 cases), and discoid lupus erythematosus (4 cases) were other common symptoms. In terms of the relationship between cutaneous symptoms and age of onset, hyper-gammaglobulinaemic purpura (mean age; 64.6 years) and blepharitis (mean age; 67.0 years) were more common in older age-onset cases. However, annular erythema (mean age; 43.1 years), subacute cutaneous lupus erythematosus (mean age; 48.4 years), and chilblains (mean age; 51.4 years) were more likely to occur among middle aged patients, indicating that the age of onset of cutaneous symptoms differed.

# RP27

## Rat model with typical cutaneous small-vessel vasculitis induced by combined injection of anti-phosphatidylserine/prothrombin complex antibody and anti-LAMP-2 antibody

Tamihiro Kawakami[1], Issei Nakade[2], Yuto Tamura[2], Fuyu Ito[3], Yuka Nishibata[2], Sakiko Masuda[2], Utano Tomaru[4], Akihiro Ishizu[2]

[1]Division of Dermatology, Tohoku Medical and Pharmaceutical University; [2]Department of Medical Laboratory Science, Faculty of Health Sciences, Hokkaido University; [3]Department of Health Protection, Graduate School of Medicine, Teikyo University; [4]Department of Pathology, Faculty of Medicine and Graduate School of Medicine, Hokkaido University, Japan

We previously reported that cutaneous vasculitis could be dependently associated with the presence of anti-PS/PT antibody and LAMP-2. Copy number of LAMP-2 mRNA in skin tissue samples from rats with cutaneous vasculitis induced by intravenous administration of anti-PS/PT antibody after subcutaneous histone injection was significantly higher than in those without cutaneous vasculitis. Male Wistar-Kyoto rats (n=14) were given a subcutaneous injection of cell-free histones on the back and then divided into four groups. Two hours later, Group 1 rats (n=4) were intravenously administered rat IgM class anti-PS/PT monoclonal antibody and anti-LAMP-2 polyclonal antibody. Group 2 rats (n=3) were given an intravenous administration of rabbit IgG instead of the anti-LAMP-2 antibody, and Group 3 rats (n=4) were given rat IgM instead of the anti-PS/PT antibody. Group 4 rats (n=3) given a subcutaneous injection of cell-free histones followed by intravenous administration of rat IgM and rabbit IgG were employed as another control. We detected typical leukocytoclastic vasculitis where neutrophils predominantly infiltrated and nuclear dust was scattered around the blood vessels in the dermis of all Group 1 rats. These observations were not evident in systemic organs other than the skin. MPO positive neutrophil infiltration in the vasculitic lesions was most abundant in Group 1 rats using immunofluorescent staining. We observed that rats in Group 2 developed cutaneous vasculitis with lymphocyte-predominant infiltration. Group 3 rats demonstrated a neutrophil recruitment into the small vessels in the skin. We suggested that the introduction of skin local histones and anti-PS/PT antibody in serum could move LAMP-2 to the cell surface of neutrophils and vascular endothelial cells, and that anti-LAMP-2 antibody could bridge these cells through antigen-specific binding in typical cutaneous small-vessel vasculitis.

# RP28

## The relationship of neutrophil extracellular traps in superficial venous thrombosis of Behcet's disease

Tamihiro Kawakami[1], Kae Yokoyama[1], Takaharu Ikeda[1], Yuka Nishibata[2], Sakiko Masuda[2], Utano Tomaru[3], Akihiro Ishizu[2]

[1]Division of Dermatology, Tohoku Medical and Pharmaceutical University; [2]Department of Medical Laboratory Science, Faculty of Health Sciences, Hokkaido University; [3]Department of Pathology, Faculty of Medicine and Graduate School of Medicine, Hokkaido University, Japan

Behçet's disease (BD) has a heterogeneous spectrum of disease manifestations featuring different organ involvement and could be characterized by different aspects in the clinical department in charge. A hierarchical clustering analysis of the hospital-based, BD was identified as five clinical independent clusters, which consist of mucocutaneous, mucocutaneous with arthritis, gastrointestinal, neurological, and ocular without other involvement subtypes. We reviewed BD patients of our hospital retrospectively and investigated neutrophils producing neutrophil extracellular traps (NETs) presence in BD patients. We recruited patients with BD (n = 12) our Dermatology Department who visited between October 2018 and September 2021. For comparison, we reviewed the records of patients with BD (n=35) from other Departments at our hospital outside of our Dermatology Department during the same period (October 2018 to September 2021). Immunolabeling of myeloperoxidase and histone citrullination proteins was performed on the skin biopsies of three BD patients who had skin biopsy-proven superficial vein thrombophlebitis in their erythema nodosum-like lesions. We observed a higher proportion of female patients, and higher incidence of acne-like eruptions among the BD patients we saw at our dermatology department, while there was a higher incidence of ocular and gastrointestinal involvement among BD patients treated in other departments. We suggest that the gender statistical trends could lead to the co-development of different manifestations, and may help clinicians to choose the best therapeutic approaches, tailoring them to a patient's specific phenotype, rather than one based on single disease manifestations. NETs were found in neutrophils of panniculitis concurrent with superficial vein thrombophlebitis. We suggest that the pathogenesis of BD-related thrombosis could be associated to neutrophil activation and NETs are released in the panniculitis of the affected skin lesions, erythema nodosum-like lesions.

# RP29

## Anti-KIF20B autoantibodies in systemic autoimmune rheumatic diseases: Their high prevalence in systemic lupus erythematosus

Haruka Koizumi[1], Yoshinao Muro[1], Satoshi Kamiya[1], Norika Akashi[1], Yuta Yamashita[1], Mariko Ogawa-Momohara[1], Takuya Takeichi[1], Marvin Flitzler[2], Masashi Akiyama[1]

[1]Department of Dermatology, Nagoya University, Japan

[2]Department of Medicine, Cumming School of Medicine, University of Calgary, Japan

**Background:** The kinesin superfamily protein 20B (KIF20B), also known as MPP-1, is a plus-end-directed motor enzyme for cytokinesis. Anti-KIF20B antibodies have been reported in idiopathic ataxia, but no previous studies have examined anti-KIF20B antibodies in systemic autoimmune rheumatic diseases (SARDs). We aimed to establish methods for detecting anti-KIF20B antibodies and to investigate the clinical significance of these antibodies in SARDs. **Methods:** Serum samples from 597 patients with various SARDs and 46 healthy controls (HCs) were included. Fifty-nine samples that had been examined by immunoprecipitation using the recombinant KIF20B protein produced by in vitro transcription/translation were used for establishing the ELISA cutoff with the same recombinant protein for measuring the anti-KIF20B antibodies. **Results:** The ELISA performed well, showing close agreement with the immunoprecipitation results (Cohen's $\kappa > 0.8$). The ELISA results for 643 samples showed the prevalence of anti-KIF20B to be higher in the SLE patients than in the healthy controls (HCs) (18/89 vs. 3/46, $p=0.045$). Since no SARD other than SLE had higher frequencies of anti-KIF20B antibodies than those of the HCs, we investigated the clinical characteristics of anti-KIF20B antibody-positive cases in SLE. The score on the SLE Disease Activity Index-2000 (SLEDAI-2K) was significantly higher for the anti-KIF20B-positive SLE patients than for the anti-KIF20B-negative SLE patients ($p=0.013$). In a multivariate regression analysis of the anti-ssDNA, anti-dsDNA, and anti-KIF20B antibodies, the presence of anti-KIF20B antibody was significantly associated with high SLEDAI-2K scores ($p=0.003$). **Conclusion:** Anti-KIF20B antibodies were found in ~20% of patients with SLE and were associated with high SLEDAI-2K scores. Much larger cohort and longitudinal studies are needed to confirm the association between anti-KIF20B antibodies and SLE.

# RP30

## Assessing prognostic factors correlating with response to nintedanib for connective tissue disease-associated interstitial lung disease: A real-world single-center study

Hiraku Kokubu[1,2], Saki Takeuchi[1], Takahisa Tozawa[1], Satoko Hisada[1], Yoshihiro Yamada[1], Yumi Itoh[1], and Masanari Kodera[1]

[1]Department of Dermatology, Japan Community Healthcare Organization Chukyo Hospital, Sanjyo, Minami-ku, Nagoya, Japan
[2]Department of Dermatology, Shiga University of Medical Science, Setatsukinowa, Otsu, Shiga, Japan

For the patients with connective tissue disease-associated interstitial lung disease (CTD-ILD), early medical intervention would be desirable. We analyze real-world single-center use of nintedanib for CTD-ILD patients. We enrolled patients with systemic sclerosis, dermatomyositis, and rheumatoid arthritis who were received on nintedanib from January 2020 to July 2022. Medical records review and stratified analysis of the data collected was conducted. Reduction of forced vital capacity in percentage of predicted (%FVC) was seen in the elderly group (>70 years, p=0.210), in male (p=0.027), the late group who started nintedanib >80 months after confirmation of an ILD disease activity (p= 0.03), the severe %DLco group (<40%, p=0.20), the group who have extensive pulmonary fibrosis at the beginning of nintedanib (pulmonary fibrosis score >35%), and the low-dose group (nintedanib 50–100 mg/day; p=0.40). %FVC did not decrease more than 5% in the young group (<55 years), the early group who started nintedanib within 10 months after confirmation of an ILD disease activity, and the group whose pulmonary fibrosis score at the beginning of nintedanib was <35%. It is important to diagnose ILD early and start antifibrotic drugs with proper timing for cases in need. We had better start nintedanib early especially for the patients who have the risks (>70 years old, male, <40 %DLco, >35% areas of pulmonary fibrosis).

# RP31

## The association between anti-U1-RNP antibodies with clinical manifestations and multiorgan damage in systemic sclerosis and systemic lupus erythematosus

Vu Huy Luong[1,2], Vu Xuan Huong[1], Le Huu Doanh[1,2], Hoang Thi Phuong[2], Do Thi Thu Hien[2]

[1]Hanoi Medical University, Vietnam

[2]National Hospital of Dermatology and Venereology, Vietnum

**Objective**: To evaluate the association between anti-U1-RNP antibodies with clinical manifestations and multiorgan damage in systemic sclerosis (SSc) and systemic lupus erythematosus (SLE). **Method**: A descriptive, cross-sectional study on 55 SSc and 48 SLE Vietnamese patients from July 2021 to September 2022. The serum anti-U1-RNP antibodies was analyzed using the immunoblot method. **Result**: A total of 55 SSc and 48 SLE patients were eligible for this analysis, among which the majority was female.. Anti-U1-RNP antibodies were present in 8 (14.5%) SSc patients and 19 (39.6%) SLE patients, mainly positive at strong level 3+. In univariate analysis, SSc patients positive for anti-U1-RNP antibodies had significantly higher prevalence of skin thickness (mRodnan 27,3 ± 6,1 vs 20,2 ± 5,8), periungual vasodilatation (87.5% vs 38.3%), periungual hemorrhage (37.5% vs 6.4%), pulmonary arterial hypertension assessed by transthoracic echocardiography (85.7% vs 34.0%) than those negative for anti-U1RNP antibodies. Meanwhile, SLE patients positive for anti-U1-RNP antibodies had also higher prevalence of Raynaud phenomenon (63.2% vs 17.2%), interstitial lung disease diagnosed by high-resolution thin-layer computed tomography (58.8% vs 23.8%), FEV1<80% (64.3% vs 30.0%). Anti-U1-RNP antibodies in SSc and SLE patients were confirmed to be independently associated with interstitial lung disease, pulmonary hypertension, muscle damage [odds ratio >1]. In addition, there was a negative association with subclinical anemia and proteinuria. **Conclusion**: In this cohort of SSc and SLE patients, anti-U1-RNP antibodies were associated with several clinical features such as skin thickness, lung, renal and hematologic disorders as well as small vasculitis.

**Keywords**: Systemic sclerosis, systemic lupus erythematosus, anti-U1-RNP, clinical manifestations, multiorgan damage.

# RP32

## Some autoantibodies in overlap syndrome of connective tissue diseases

Hoang Thi Phuong[1], Le Huu Doanh[1,2], Le Huyen My[1], Vu Huy Luong[1,2], Tran Hau Khang[2]

[1]National Hospital of Dermatology and Venereology, Hanoi, Vietnam
[2]Hanoi Medical University, Hanoi, Vietnam

The study was performed on 63 Overlap syndrome patients at NHDV from July 2019 to August 2020. **Aim**: to evaluate incident of some autoantibodies in overlap syndrome of connective tissue diseases. **Result**: 84,5% of the patients was female, the most common Overlap type is SSc/SLE (39,7%) and SSc/DM. ANA is the most prevalent autoantibody (98,4%), then anti RNP/Sm (42,9%), anti Ro-52 (42,9%), anti Ku (27%) and anti PM/Scl (11,1%). As for the autoantibodies detected in clinical variants of Overlap syndrome, anti SSA (32%), anti Ro-52 (56%) and anti RNP/Sm are mostly found in SSc/SLE; anti Ku (45,5%) and anti PM/Scl-75 (18.,2%) in SLE/DM; and anti Scl-70 (31,8%) and anti PM/Scl-100 (13,6%) in SSc/DM.

# RP33

## FACTORS ASSOCIATED WITH DIAGNOSTIC DELAYS IN DERMATOMYOSITIS

Lindsey J Wanberg[1], Nawang Singhe[1], Cody J Rasner[1], Joseph McGrath[1], Connor R Buechler[1,2,3], David R Pearson[1,2]

[1]University of Minnesota Medical School; [2]Department of Dermatology; [3]Department of Internal Medicine, USA

**Background**

Dermatomyositis (DM) is an autoimmune disease that necessitates prompt diagnosis due to associated morbidity, mortality, and risk of underlying malignancy. However, patients with DM often experience diagnostic delays (DDs) due to diverse clinical manifestations, nonspecific histopathology and biomarkers, and imperfect diagnostic criteria. We aim to characterize DDs in DM and determine how DDs are affected by demographic and disease factors.

**Methods**

Retrospective cohort study of adult patients with DM at an academic, tertiary referral center in a metropolitan area. After identification by ICD codes, charts were reviewed to determine dates of patient-reported symptom onset (SO), initial presentation to a health care provider (IP), and diagnosis of DM. DD was defined as the time between SO and diagnosis. The data were evaluated by median (IQR) and Wilcoxon rank sum testing.

**Results**

223 patients met inclusion criteria. Median DD was 7.4mo (3.1-17.2), with 20.8% experiencing DD ≥24mo. DDs did not differ by sex or race, but patients ≥60y had shorter DDs ($p=0.003$). Patients with classic DM had shorter DDs than those with clinically amyopathic DM ($p=0.005$), and paraneoplastic DM was diagnosed faster than non-paraneoplastic DM ($p=0.023$). Patients presented a median of 1.8mo (0.7-5.2) after SO. 79.4% had a rash at IP and 32.7% had both rash and muscle symptoms. Although muscle symptoms at IP predicted shorter DDs ($p=0.005$), rash did not ($p=0.958$).

**Conclusion**

Patients with DM experience substantial DDs, which may result in disease progression and later detection of malignancy. Patients ≥60y and those with muscle involvement had shorter DDs. Even though rash was the most common symptom at IP, this did not result in earlier diagnosis, underscoring the difficulty of DM rash identification. To improve DDs, providers across specialties should be aware of the common distribution and appearance of DM rash and increase vigilance for disease onset in younger patients.

# RP34

## Immunohistological pattern-stratification opens the door for reliable targeted treatment strategies in cutaneous lupus erythematosus

Joerg Wenzel

University Hospital Bonn, Germany

Background: New developments in immunohistological (IHC) and molecular pattern analyses of cutaneous lupus erythematous (CLE) skin lesions have provided significant insides into the inflammatory pathways activated in this autoimmune disorder. In parallel, progress in the design of targeted therapies generated a spectrum of different specific tools for therapeutic intervention. Aim of this study was to develop an immunohistological algorithm to identify patients prone to specific targeted therapies.

Patients and methods: Skin biopsies of CLE patients ($N = 119$, subtypes: subacute cutaneous LE, chronic discoid LE, chilblain LE, LE tumidus) were screened for the expression of surrogate markers for targeted therapies available and/or in clinical studies for CLE (MxA = type I/III interferons; CD20 = B cells; CD123 = plasmacytoid dendritic cells).

Results: This IHC-screening algorithm identified 92% (n=109) patients who expressed at least one on these IHC-markers. 28% expressed all 3 markers at the same time, 17% MxA & CD20, 5% CD123 & CD20, 9% CD123 & MxA, 18% MxA, 7% CD20 and 9% CD123.

Discussion: This study demonstrates that > 90% of CLE patients might be prone to be treated with at least one targeted drug, which were developed for the treatment of SLE or is in clinical studies for CLE. These drugs include belimumab (anti-Blys/ B cells) and anifrolumab (anti-IFNabR), which have already been approved for the treatment of SLE by FDA and EMA, but also anti-pDCs drugs (BIIB059 (anti-BDCA2); Daxdilimab (Anti-ILT7)) and inhibitors of the JAK/STAT pathway (Tofacitinib (JAK), Lanraplenib & Filgotinib (JAK/SYK), Deucravacitinib (TYK2), which block IFN-mediated inflammatory pathways. Further studies will provide more data if an IHC-based pre-treatment stratification of our patients really can help to identify ideal drugs for individual patients and to reduce the number of non-responders in clinical practice.

# CP1

## A new autoantibody screening and quantification system with wet protein arrays detected anti-Sjögren's syndrome/scleroderma autoantigen 1 (SSSCA1) antibody in a patient with very early systemic sclerosis

Yuta Norimatsu, Taro Akatsuka, Akari Matsuoka, Yuka Sakai, Sohshi Morimura, Toshihisa Hamada, Makoto Sugaya

Department of Dermatology, International University of Health and Welfare, Japan

Systemic sclerosis (SSc) is a chronic autoimmune disease characterized by vascular injury and fibrosis of the skin and internal organs. Although specific autoantibodies are supposed to be involved in the pathogenesis of SSc, most of them cannot be detected by the tests that the health insurance covers. Recently, the autoantibody screening and quantification with wet protein arrays called A-cube$^{TM}$ were developed in Japan. The assay can simultaneously measure 65 types of specific autoantibodies for SSc and dermatomyositis, using only 300 μL of serum. Here we report a case with very early SSc with anti-Sjögren's syndrome/scleroderma autoantigen 1 (SSSCA1) antibody, which was detected by the new assay. A 49-year-old woman presented to our hospital in May 2022 with a chief complaint of persistent frostbite. Her medical history included panic disorder and reflux esophagitis. Her mother had SSc with anti-centromere antibody. The patient had Raynaud's symptoms, nail fold bleeding, and vascular abnormalities of the posterior nail cords, but no swelling or hardening of the fingers. Antinuclear antibodies were positive and she was diagnosed with very early SSc. Systemic examination revealed no evidence of cardiac disease, interstitial lung disease, or malignancy. The results of tear and saliva ferning tests denied Sjögren's syndrome. The new autoantibody screening found that her serum was positive for anti-SSSCA1 antibody. SSc patients with this antibody are reported to have a higher risk of severe Raynaud's symptoms, cardiac diseases, and malignancy. We plan to follow up the patient carefully, although she currently does not have these complications.

# CP2

## Multiple peculiar mucinous nodules on the during over corticosteroid treatment in a patient with SLE

TAKASHI ITO[1], YUKA HANAMI[1], TOSHIYUKI YAMAMOTO[1],

[1]Fukushima Medical University, Japan

We here report a case of systemic lupus erythematosus (SLE) with multiple peculiar nodules in a 45-years-old; female patient. Seven months before, she had marked thrombocytopenia, and treatment with steroids was started based on a diagnosis of idiopathic thrombocytopenia at the hematology department of another hospital. Two months later, she developed severe nephrotic syndrome and progressive renal dysfunction, and was referred to the nephrology department of our hospital. Lupus nephritis associated with SLE was suspected and referred to our department. Around the same time as the onset of nephrotic syndrome, she developed multiple nodules without spontaneous symptoms, mainly on her upper extremities. At the time of her first visit to our department, she had a mildly sunken brownish nodule about 1 cm in diameter with a yellowish rim and a soft swelling surrounding the nodule. A few similar nodules were seen in the lower extremities. Histopathology showed proliferation of fibroblast-like cells in the upper dermis as well as perivascular lymphocyte infiltration in the superficial dermis in the central part of the nodule. At the margin of the eruption, mucin deposition in the interstitium was observed from the superficial layer to the deep layer of the dermis.

# CP3

## Ultrasound Visualizes Peripheral Vascular Dysfunction in Finger Pulp Blood Flow in Patients with Systemic Sclerosis

Kazuhiro Komura[1], Minoru Hasegawa[2]

[1]Department of Dermatology, Kanazawa Red Cross Hospital, Japanese Red Cross Society
[2]Department of Dermatology, University of Fukui, Japan

There are no reliable biomarkers of vascular severity or extension, so identification of patients with disabling or life-threatening vascular involvement is often difficult and too late. Best therapeutic effects include healing of ulcers and improvement of blood flow in pulmonary, renal and peripheral vascular beds in systemic sclerosis (SSc). Recent advance of medical devices enables precise recognition of small architectures, so that peripheral blood flow is used for one of individual identifications. Herein, we have demonstrated 2D and 3D color doppler ultrasonography (Aplio i700 TUS-AI700E, Canon medical systems corporation, Japan) images of peripheral blood perfusion on distal phalanx of third finger from two patients with SSc. The diagnosis of SSc was made according to 2010 Diagnostic criteria for SSc: From the Japan Scleroderma Study Conference.

The patients did not have any experience of digital ulcers. Evaluation was performed in baseline conditions after 30 min staying at air-conditioning room (24 °C). The images were processed using ImageJ software. Pixels within the threshold limit allowed a distinction with fair accuracy between the blood flow signal and the background signal. After applying the threshold settings, image analysis was used to measure the total area. The signals in SSc were markedly reduced (as <5% healthy control) within dualized 2D ultrasound color doppler images, which were supported by 3D structure. Thus, ultrasound may have a potential to describe peripheral vascular architectures in early SSc, although further analysis is needed.

# CP4

## Cutaneous lupus erythematosus with negative for serological findings and direct immunofluorescence: How would you diagnose?

Daisuke Suzuki

Department of Dermatology, International University of Health and Welfare, Japan

Cutaneous lupus erythematosus (CLE) is a connective tissue disease with varying clinical presentations. CLE can occur as its own entity or in conjunction with systemic lupus erythematosus (SLE). Some patients have skin-limited disease, others develop systemic symptoms and subsequently progress to SLE. Therefore, the diagnosis of CLE is very meaningful; we can provide a suitable treatment for skin-limited CLE patients and an appropriate evaluation of the disease activity for SLE patients. However, the diagnosis of CLE is challenging actually. Although sometimes serological findings or direct immunofluorescence are helpful, the diagnosis of CLE is often based on the clinical and histopathological features and requires high expertise. I herein report a case of CLE with negative for serological findings and direct immunofluorescence. A 69-year-old Japanese female patient presented with facial erythema, for which topical corticosteroids were not effective. A physical examination showed poorly-demarcated and indurated erythematous plaques on her cheeks and neck. No autoantibodies were detected in serum examination. A histopathological examination revealed patchy superficial and deep lymphocytic infiltrate around adnexae and vessels with faint vacuolar degeneration of dermo-epidermal junction. Direct immunofluorescence was revealed no depositions of IgG, IgA and IgM on the basement membrane zone. She was diagnosed with CLE and treated with topical calcineurin inhibitors (0.1% tacrolimus ointment). However, because of repeated improvement and worsening of symptoms, she was subsequently treated with hydroxychloroquine (HCQ) in addition. Her skin lesions improved gradually, and finally almost disappeared. The fact that HCQ was so effective for her skin lesions confirmed her diagnosis of CLE. In this case, I suspected CLE from clinical findings and gave her a diagnosis of CLE based primarily on histopathological findings. How would you diagnose such a case?
